# Supplementary material for: SAVANA: reliable analysis of somatic structural variants and copy number aberrations using long-read sequencing
Source: Nat Methods. 2025 May 28;22(7):1436–46. doi: 10.1038/s41592-025-02708-0 (PMC12240814; doi:10.1038/s41592-025-02708-0)
Supplement: Supplementary file 1 — Supplementary Figs. 1–37. [file 41592_2025_2708_MOESM1_ESM.pdf]

nature methods

Supplementary information https://doi.org/10.1038/s41592-025-02708-0

SAVANA: reliable analysis of somatic structural variants and copy number aberrations using long-read sequencing

In the format provided by the authors and unedited

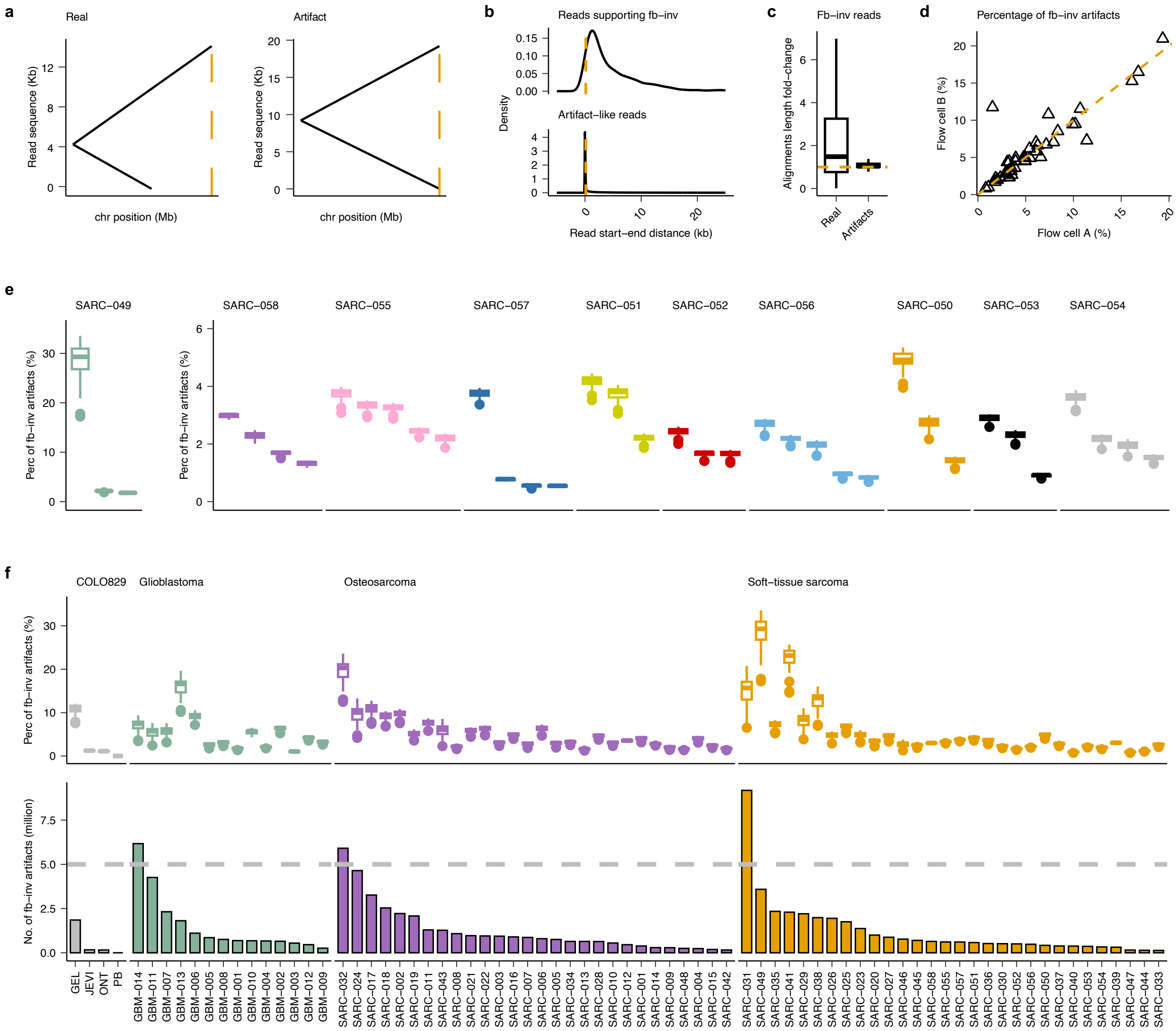

Supplementary Figure 1. Analysis of fold-back-like inversion artifacts. (**a**) Schematic representation of a read alignment supporting a real fold-back inversion (left) and a fold-back-like inversion artifact (right). The dashed yellow line indicates the end alignment position in each case. (**b**) Distribution of the distance between the read start and end alignment positions. Although a uniform distribution is expected for *bona fide* fold-back inversion events, a peak is observed around 0, indicating the presence of fold-back-like inversion artifacts. A threshold of 150bp was selected to identify fold-back-like inversion artifacts. (**c**) Boxplot showing the fold-change of the forward and reverse alignment lengths. For reads supporting artifactual fold-back-like inversions, the forward and reverse alignment lengths are equal, resulting in a fold-change clustering around one. (**d**) Scatter plot showing the correlation of the percentage of artifacts between flow cell replicates. The dashed yellow line corresponds to the diagonal. (**e**) Boxplot showing the percentage of artifacts per chromosome across different tumour regions and patients. The differences are greater among regions than chromosomes, further supporting the artifactual origin of fold-back-like inversions. (**f**) Percentage of fold-back-like inversion artifacts per chromosome (upper panel) and the total number of fold-back-like inversion artifacts per tumour (lower panel) across the study cohort. For visualization purposes, the tumour region with the highest rate of artifacts was selected as the representative sample for each patient. The dashed grey line indicates the threshold of 5 million fold-back-like inversion artifacts. Fb-inv: fold-back inversion; Perc: percentage.

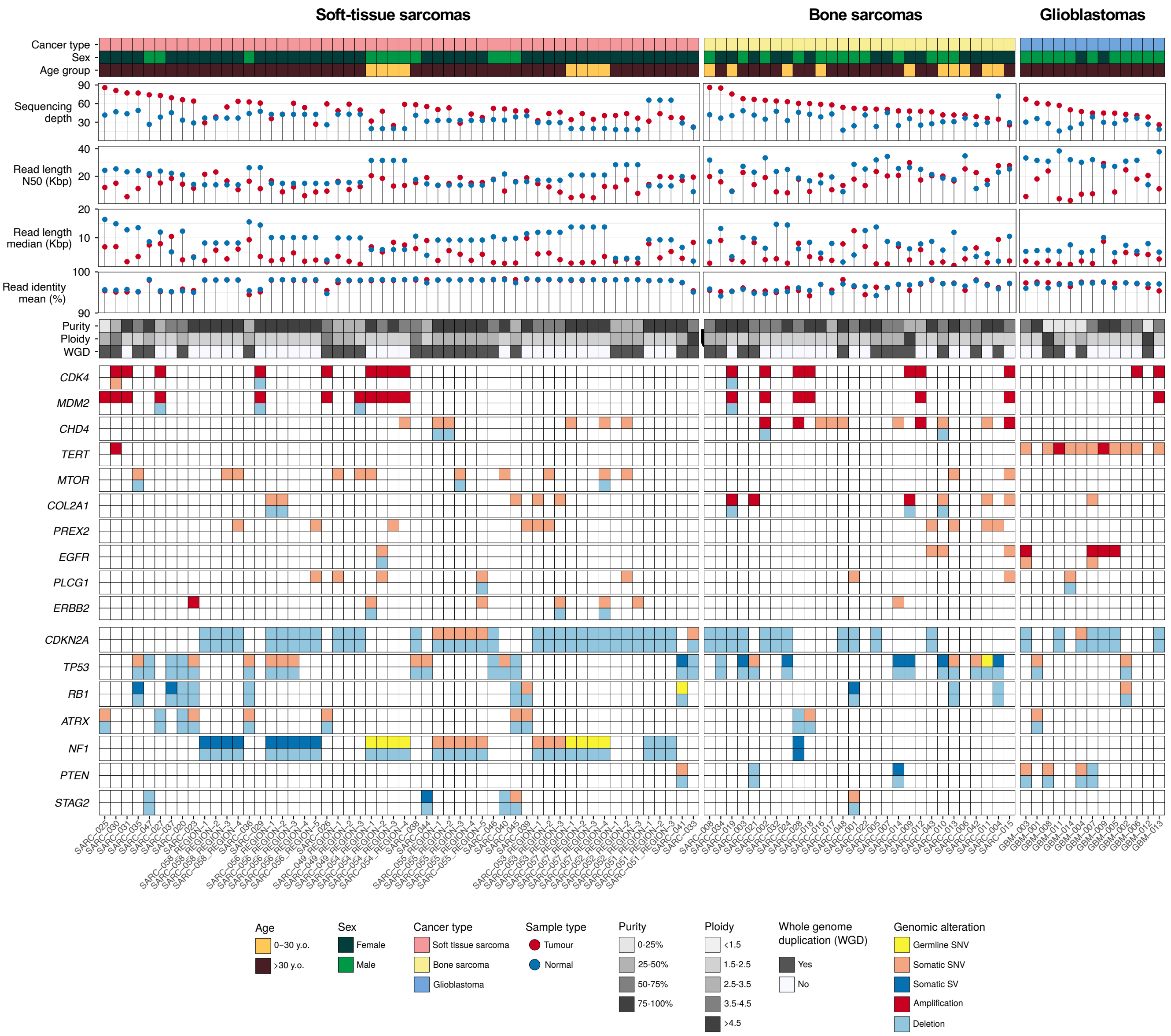

Supplementary Figure 2. Overview of the tumour samples analysed in this study. Genomic and clinical landscape of the tumour samples analysed in this study using matched nanopore and Illumina WGS. Clinical information, histopathologic features, biallelic mutations and amplification of oncogenes are shown. The lollipop plots show the nanopore sequencing run results for the tumour and matched whole-blood samples. For *TERT*, only activating hotspot promoter mutations (c.-124C>T and c.-146C>T) were considered.
Kbp: kilobase pairs; WGD: whole-genome doubling.

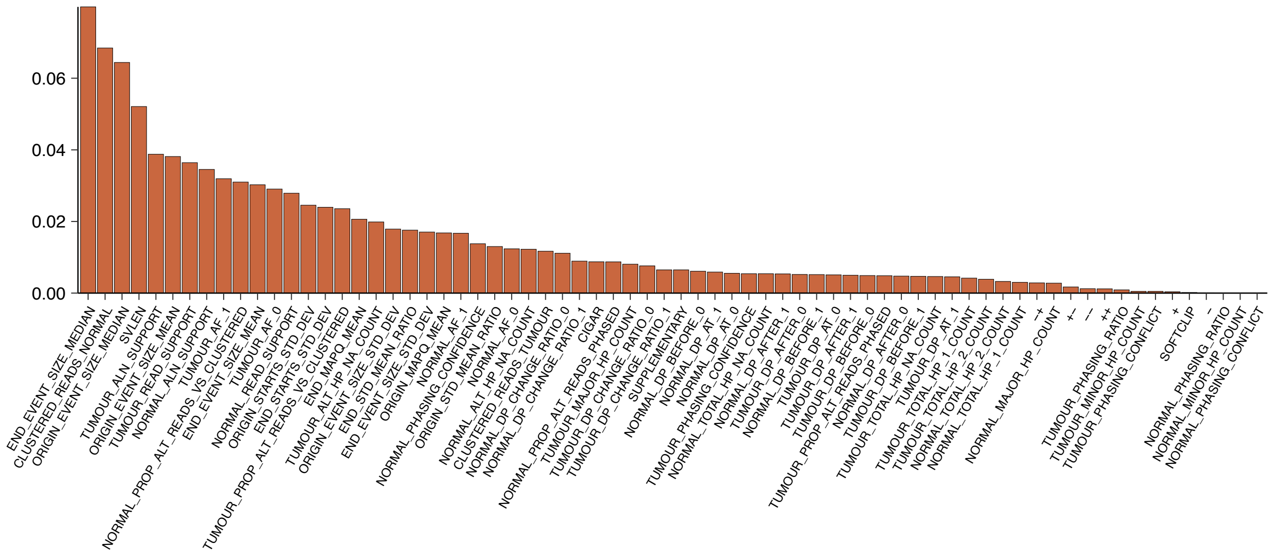

Supplementary Figure. 3. Mean decrease in accuracy (feature importance) obtained for each variable used in the Random Forest model to distinguish sequencing artefacts from true somatic SVs. A description for each feature is provided in Supplementary Table 2.

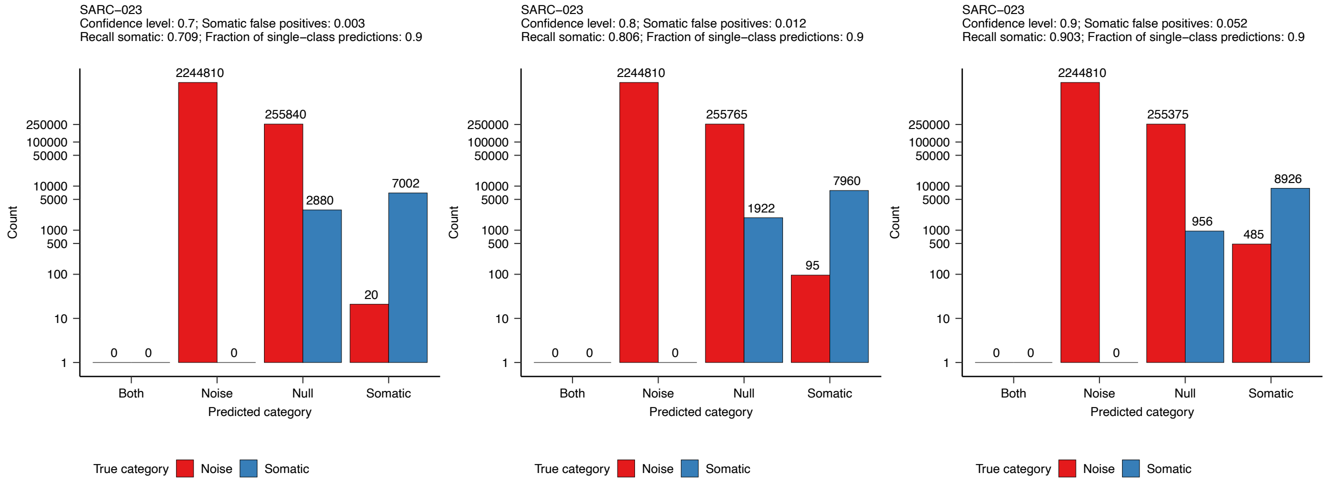

Supplementary Figure 4. Analysis of the predicted categories using Mondrian Conformal Prediction. The bars show the number of breakpoints predicted as true somatic SVs in blue, whereas red bars represent the number of breakpoints predicted to as sequencing or mapping artefacts as a function of the confidence level.

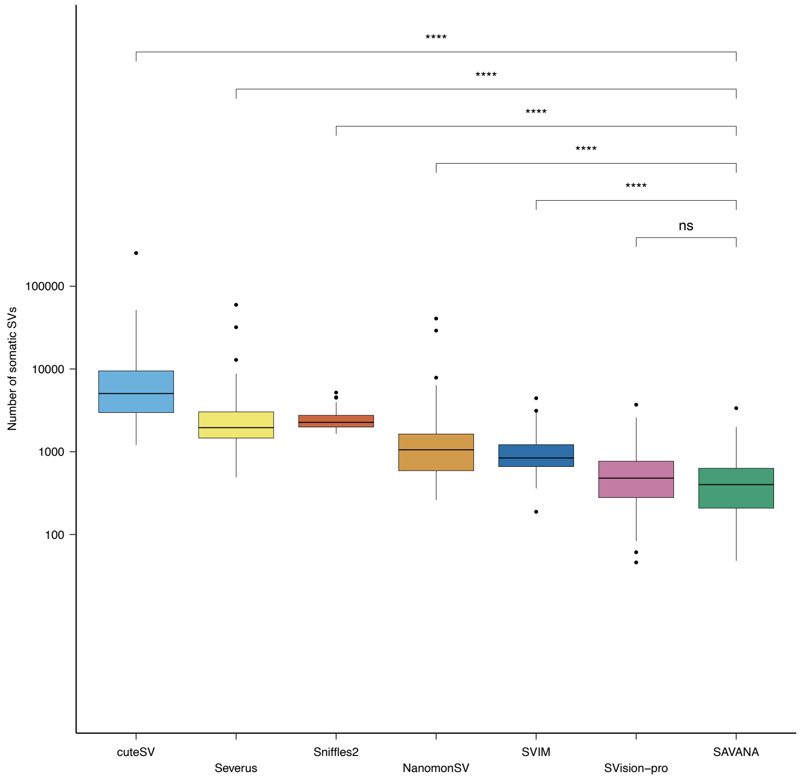

Supplementary Figure 5. Total number of somatic SVs detected across the cohort using the SV detection algorithms benchmarked in this study. Significance was assessed using the two-sided Wilcoxon's rank test; $**P < 0.001$, $****P < 0.00001$.

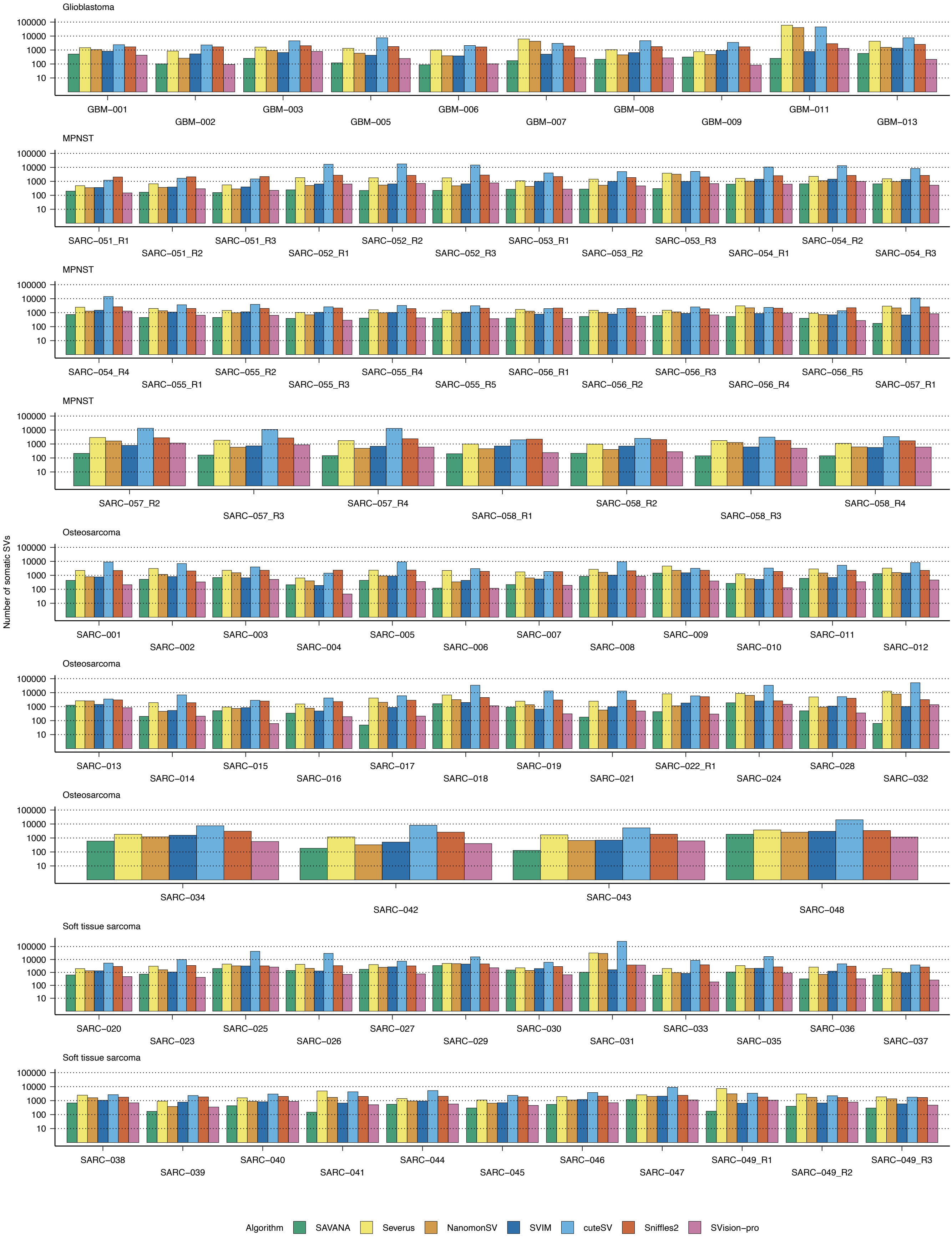

Supplementary Figure 6. Total number of somatic SVs detected in each sample by the algorithms benchmarked.

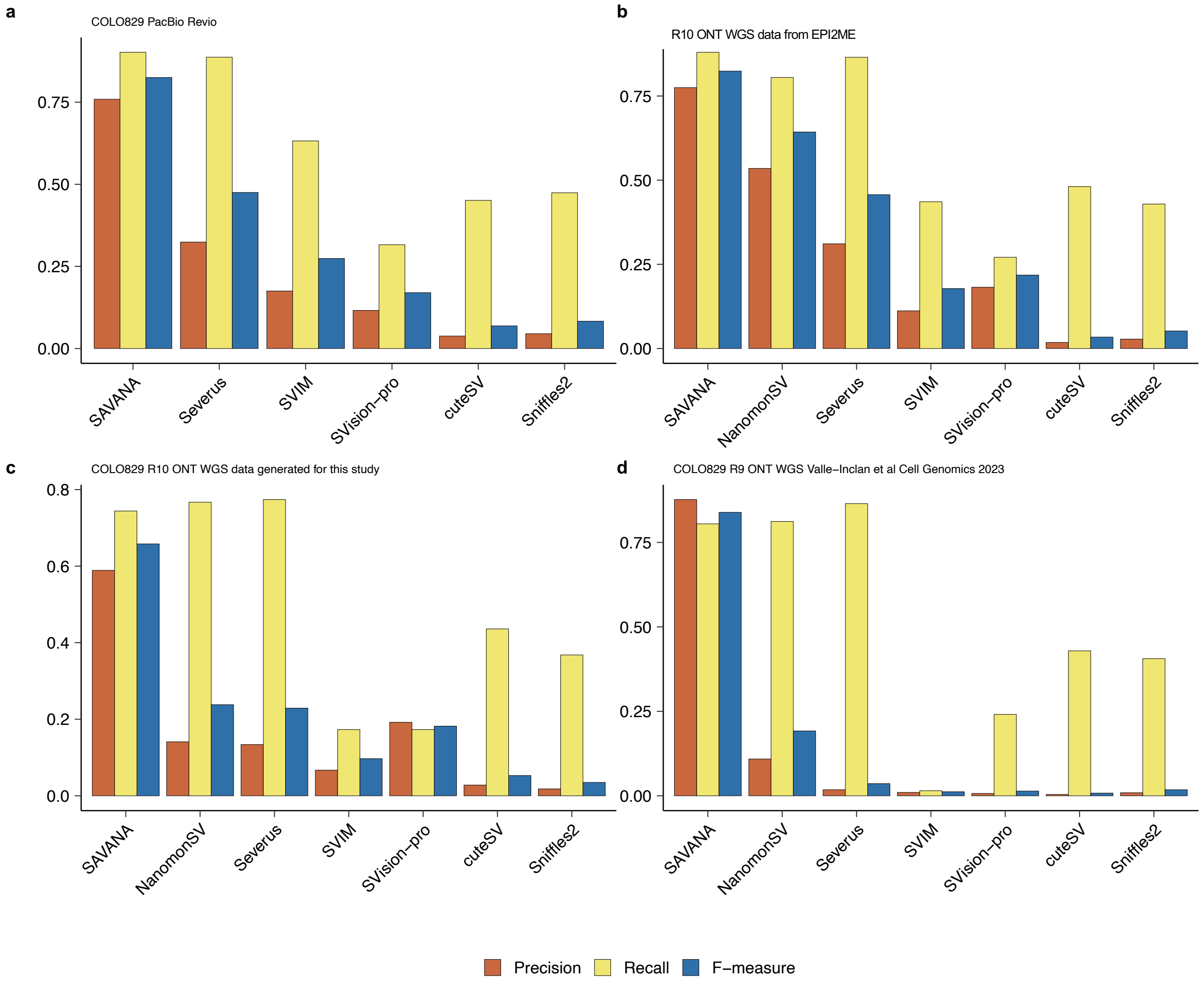

Supplementary Figure 7. Benchmarking of SAVANA against existing SV detection algorithms using a truth set of 68 somatic SVs detected in the melanoma cell line COL0829 and its matched normal cell line COLO829BL. Benchmarking results using (**a**) WGS data generated using the Revio platform from PacBio; (**b**) nanopore WGS data generated by Oxford Nanopore Technologies using R10 PromethION flow cells; (**c**) nanopore WGS data generated for this study using R10 PromethION flow cells; and (**d**) nanopore WGS data generated by Valle-Inclán et al. Cell Genomics (2022) using R9 PromethION flow cells.

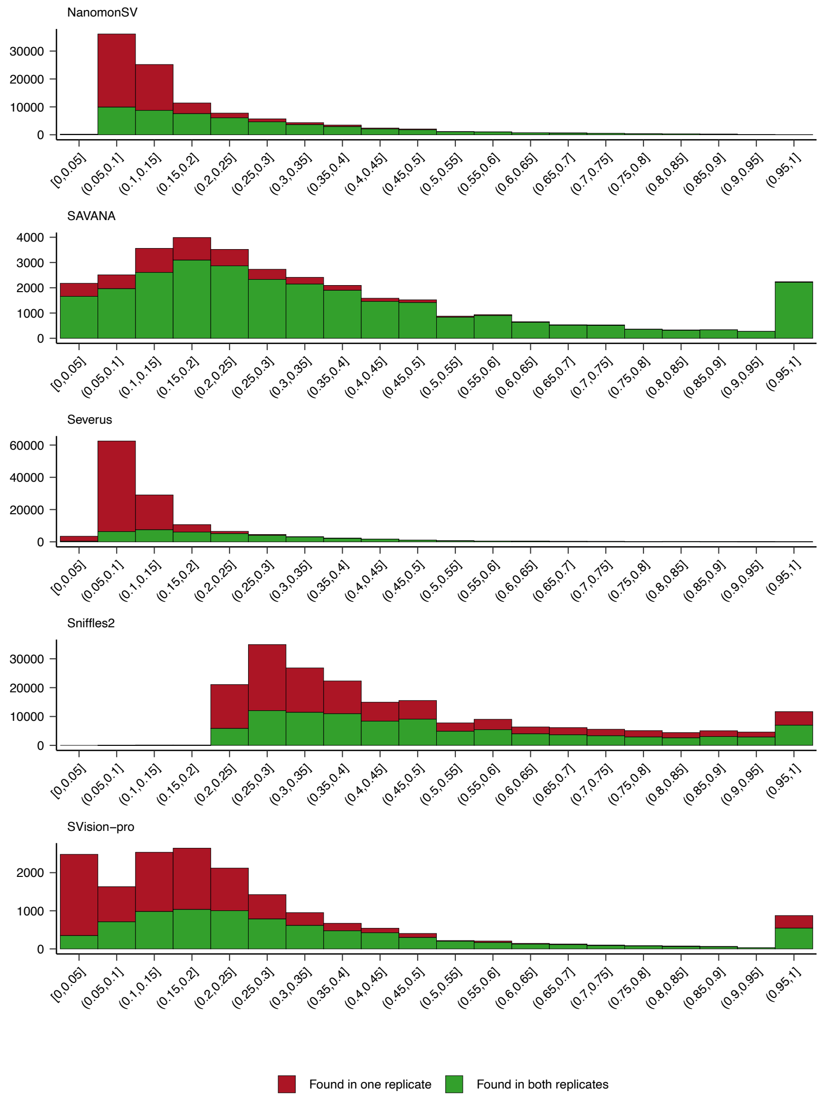

Supplementary Figure 8. Comparison of SVs detected in one or both replicates as a function of the SV allele fraction. Comparison of the count of somatic SVs detected in one (red) or both (green) replicates stratified by allele fraction. Note different y-axis scales used to reflect the number of SVs reported by each algorithm. Only algorithms that report the allele fraction or information which can be used to calculate the allele fraction of SVs were included in this analysis.

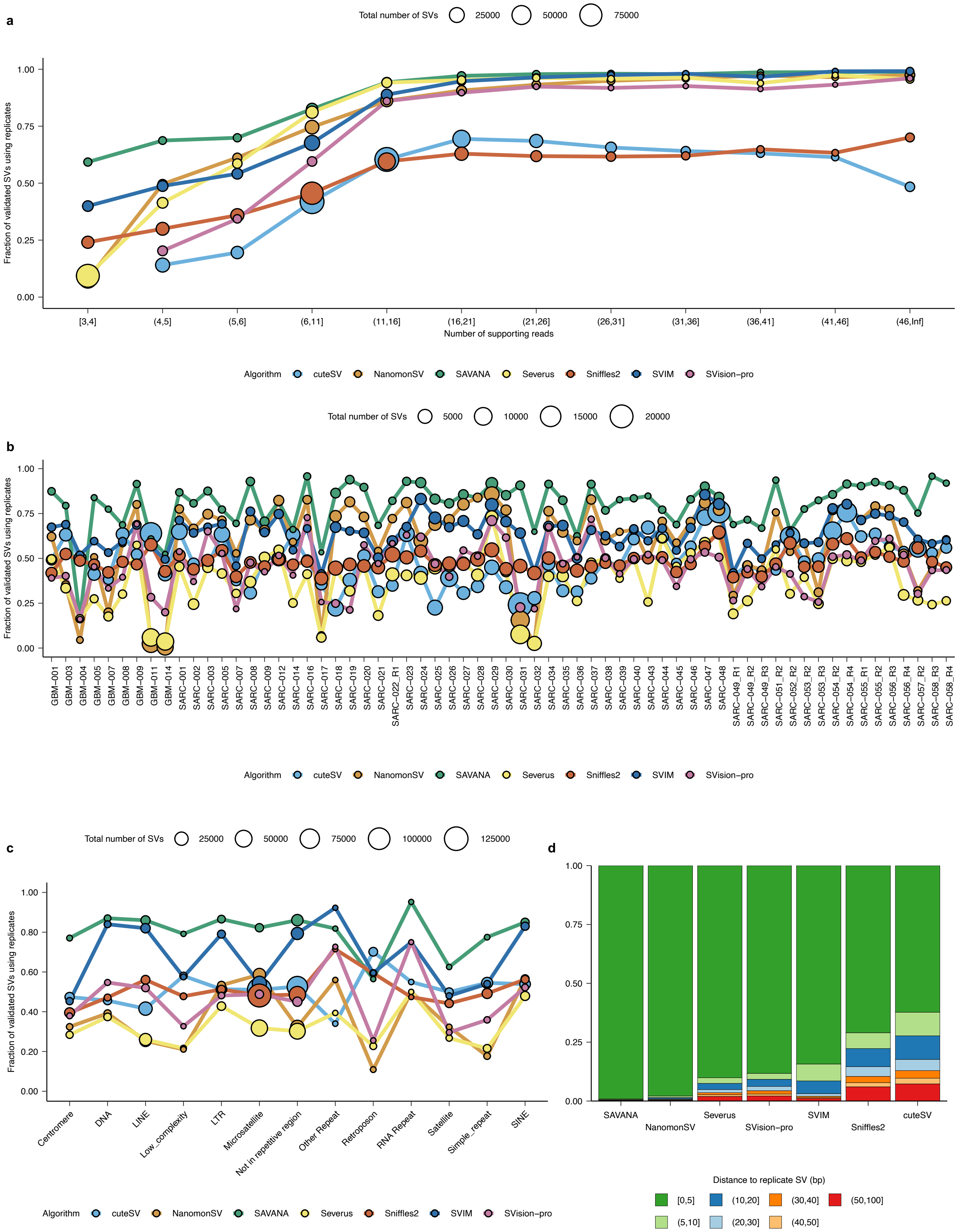

Supplementary Figure 9. Total number of somatic SVs detected in each sample.
(**a**) Fraction of somatic SVs detected in both replicates as a function of the number of reads supporting each SV. (**b**) Fraction of somatic SVs detected in both replicates for each sample. (**c**) Fraction of somatic SVs detected in both replicates across repetitive regions in the genome (GRCh38). (**d**) Replicated SVs coloured by the distance to their matching replicate SV in bps. The size of the dots in **a-c** represents the number of somatic SVs in each group. Panels **a-c** show the aggregated results for the 64 samples with the highest sequencing depth.

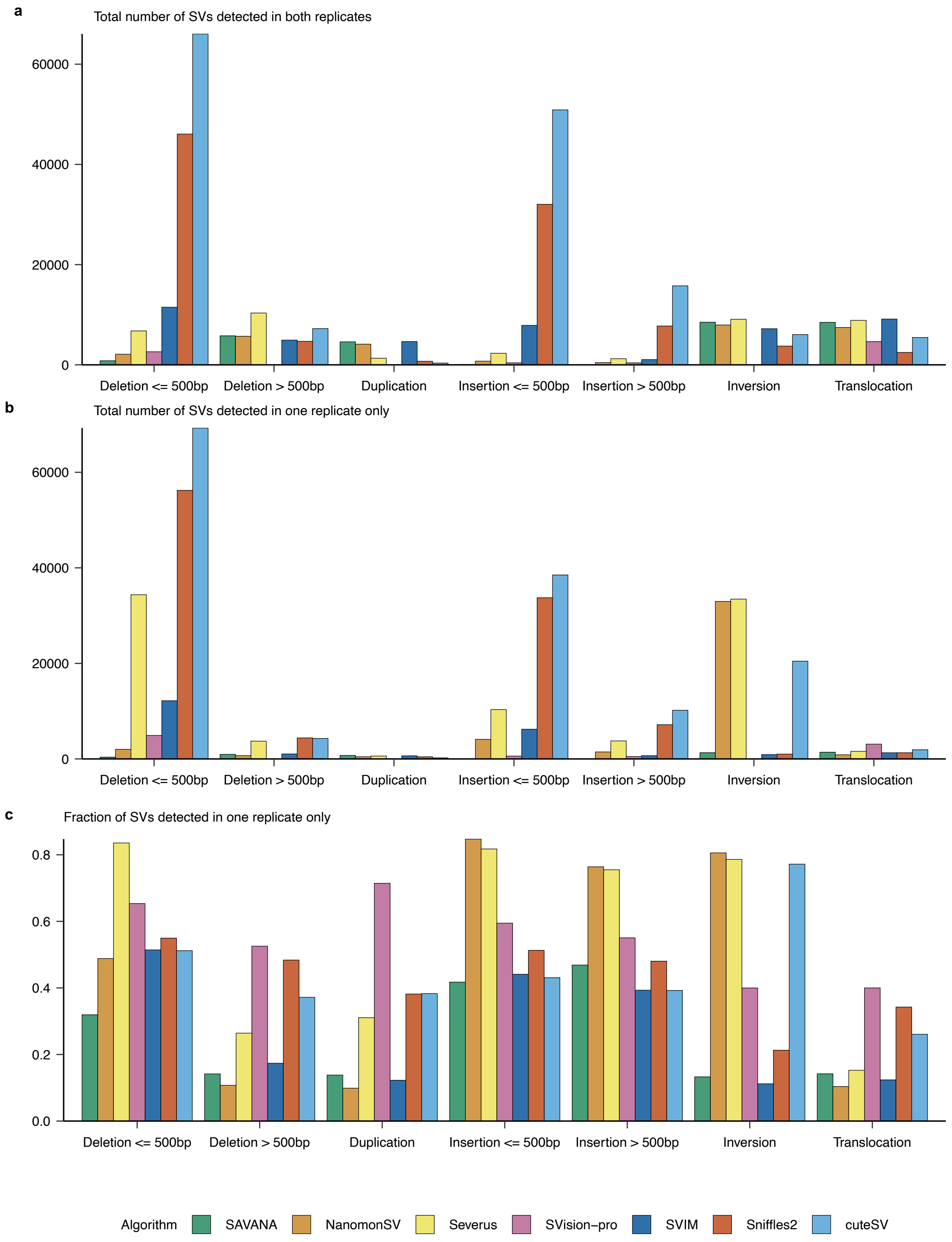

Supplementary Figure 10. Analysis of somatic SVs detected across replicates stratified based on SV type.
(**a**) Total number of SVs detected in all replicates. Fraction (**b**) and total number of SVs (**c**) detected in one replicate only. The bars in **a-c** show the aggregated data across all tumours analysed.

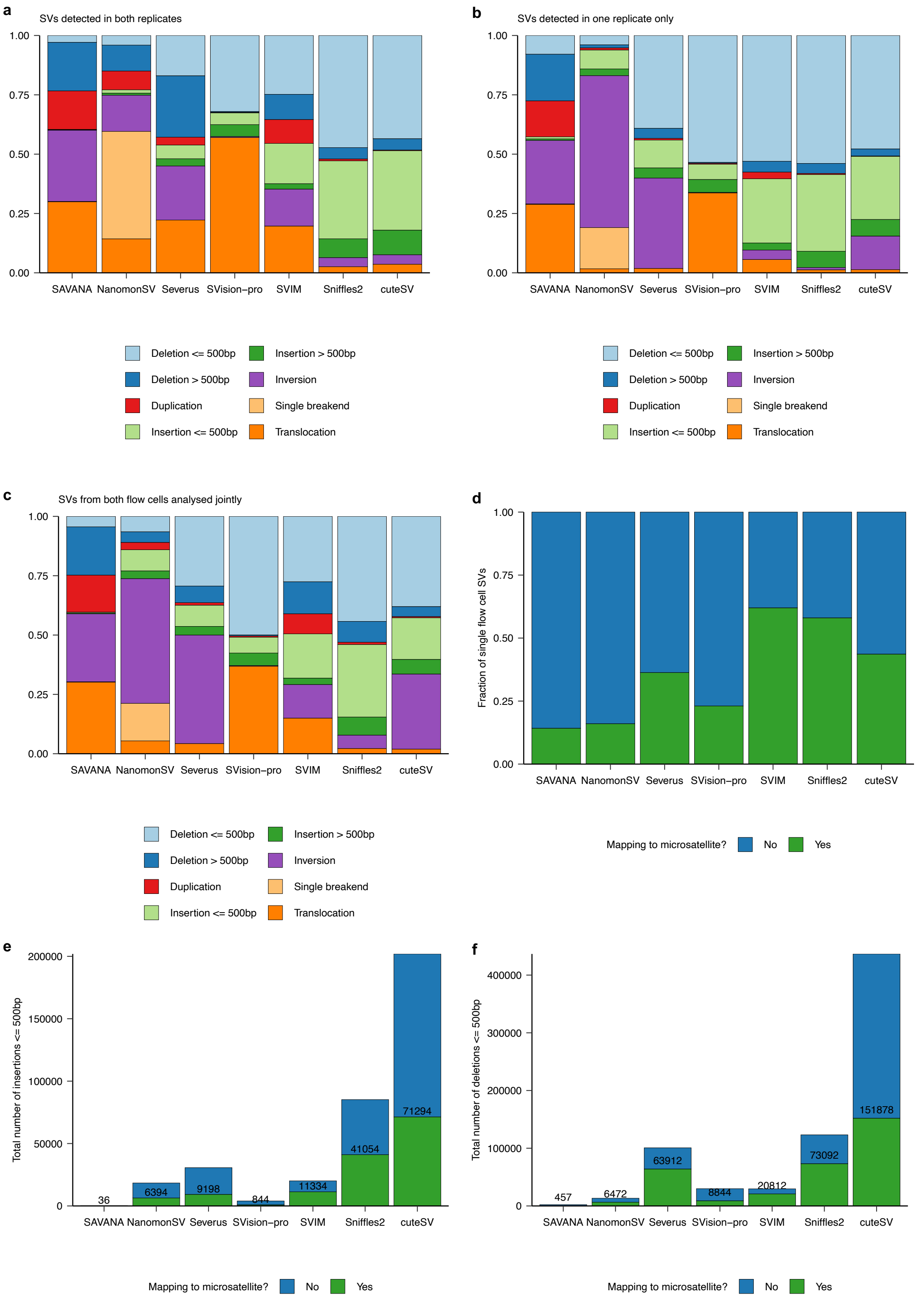

Supplementary Figure 11. Analysis of the types of somatic SVs detected by the SV detection algorithms benchmarked. (**a**) Fraction of SVs detected in both replicates stratified based on the type of SV. (**b**) Fraction of SVs detected in one replicate only stratified based on the type of SV. (**c**) Distribution of SV types detected when the sequencing data from both flow cells was analysed jointly. (**d**) Fraction of SVs detected in one replicate only that mapped to microsatellite loci. Total number of insertions (**e**) and deletions (**f**) detected across all tumours when the sequencing data from all flow cells used to sequence each tumour sample were analysed jointly. The numbers on top of the bars in **e-f** indicate the total number of insertions and deletions, respectively, that mapped to microsatellite loci across all tumours analysed.

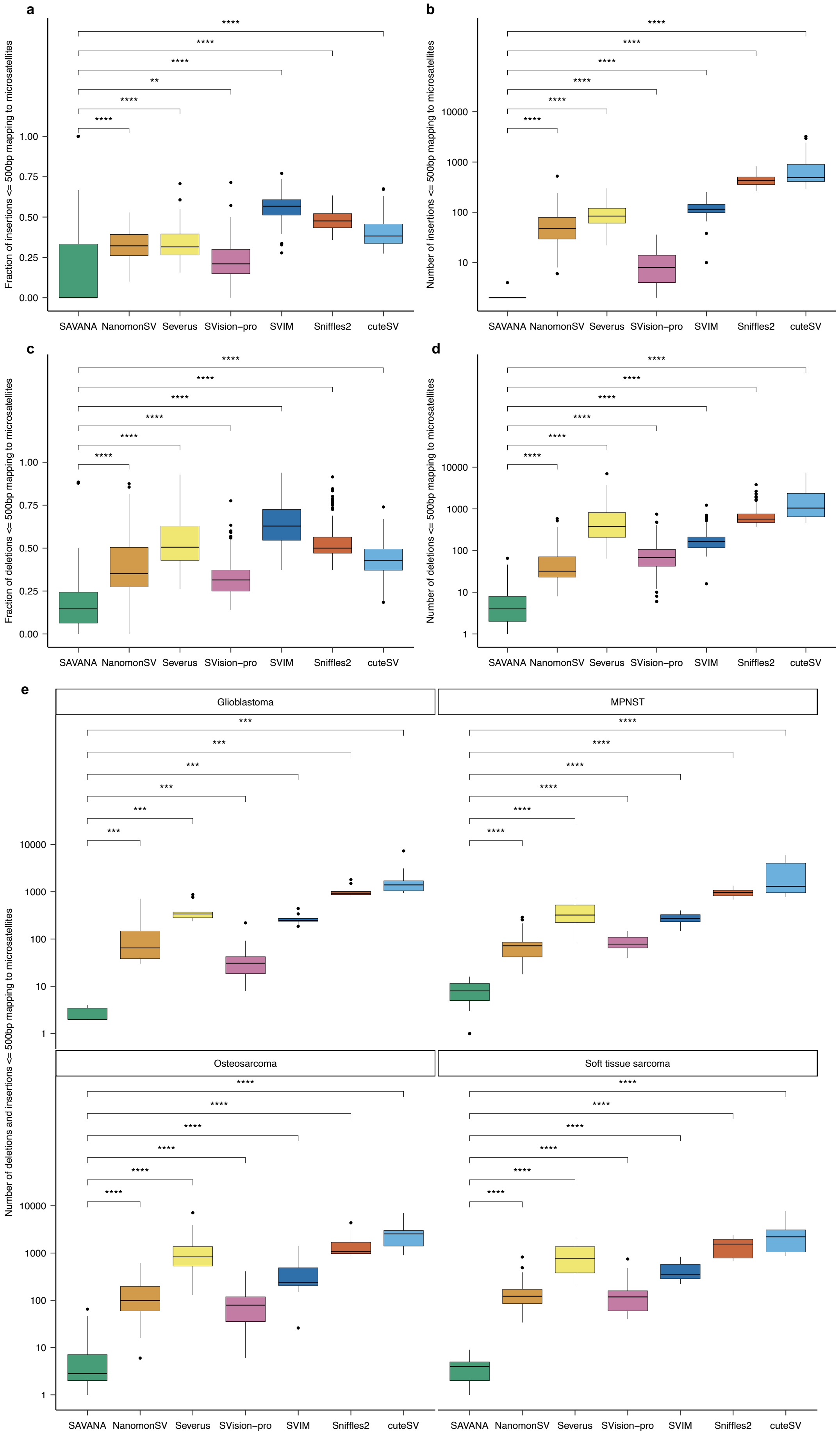

Supplementary Figure 12. Analysis of the genomic distribution of deletions and insertions detected when analysing the tumour sequencing data from both flow cells jointly.
Fraction (**a**) and total number (**b**) of insertions detected across all tumours analysed mapping to microsatellite loci. (**c**) Fraction of deletions smaller than 500bp detected across all tumours analysed mapping to microsatellite loci. (**d**) Fraction of deletions and insertions detected across all tumours analysed mapping to microsatellite loci. (**e**) Analysis of the fraction of deletions and insertions mapping to microsatellite loci stratified based on tumour type: glioblastomas, malignant peripheral nerve sheath tumours (MPNSTs), osteosarcomas, and soft-tissue sarcomas other than MPNSTs.

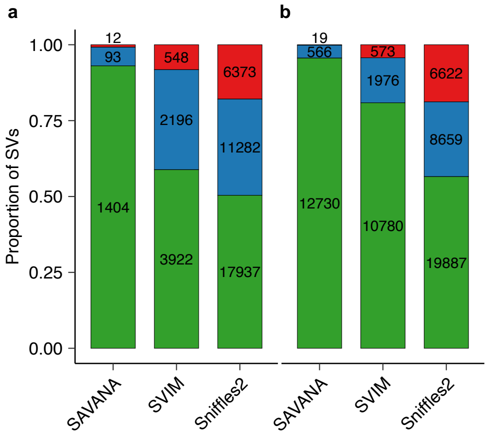

Supplementary Figure 13. Detection of false positive calls among the SVs called in one (a) or both replicates (b) using read-backed phasing. SVs across the cohort (64 tumour-samples split into two replicates each) supported by sequencing reads assigned to only one parental allele are coloured in green. SVs with significant read support from both parental alleles are shown in red, and those with inconclusive results are shown in blue. Overall, somatic SVs called by SAVANA in both replicates are primarily supported by sequencing reads from a single parental allele, whereas SVs detected by other methods in either one or both replicates are supported by sequencing reads from both parental alleles, consistent with them being false positive SV calls.

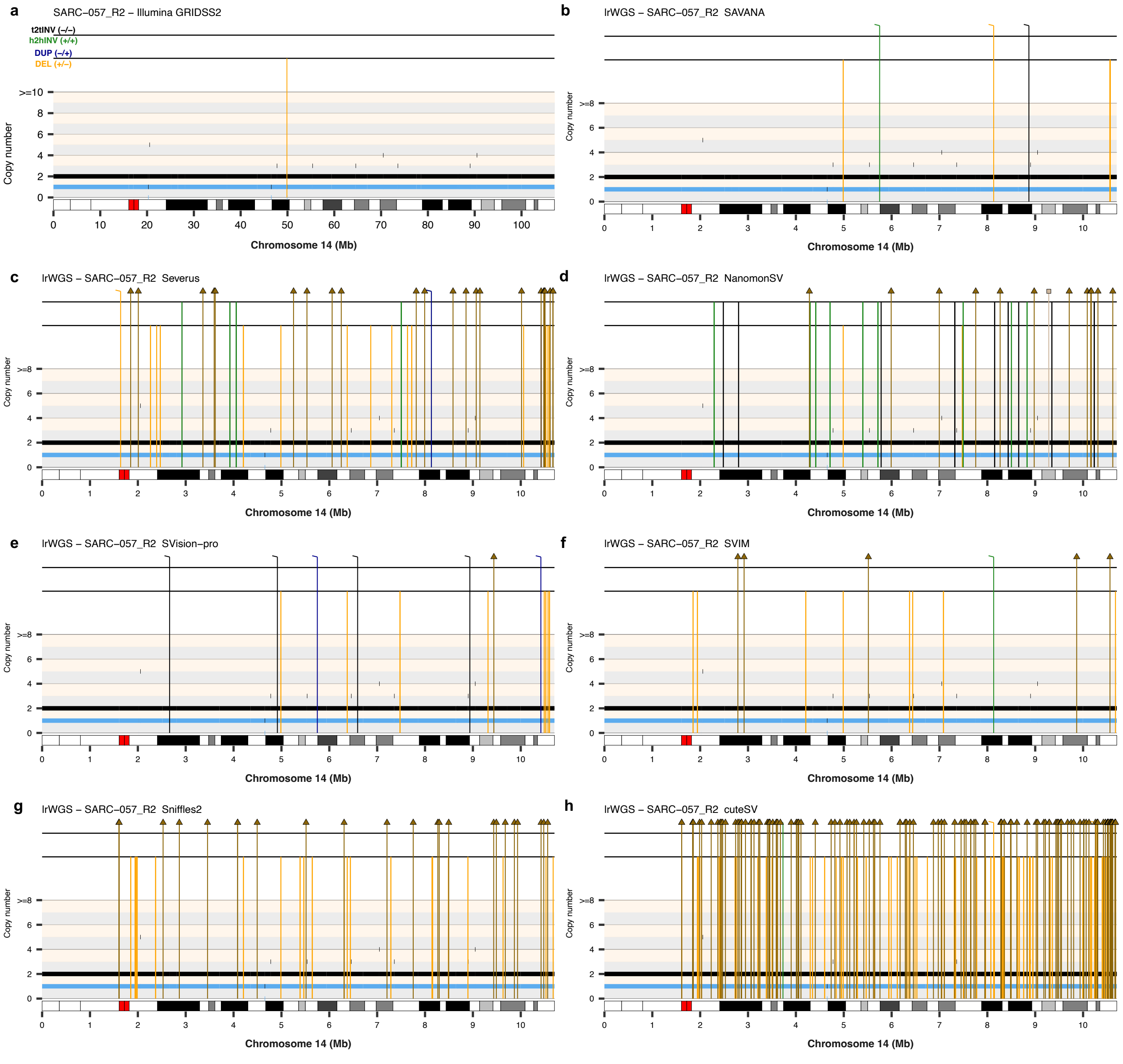

Supplementary Figure 14. Comparison of the SVs detected in tumour region 2 from the MPNST SARC-057. (**a**) Somatic SVs and copy number profiles detected using GRIDSS2 and PURPLE in whole-genome short-read sequencing data. Somatic SVs detected in matched long-read nanopore whole-genome sequencing data (lrWGS) using SAVANA (**b**), Severus (**c**), NanomonSV (**d**), SVision-pro (**e**), SVIM (**f**), Sniffles2 (**g**) and cuteSV (**h**). The copy number profiles shown in **a-h** were calculated using PURPLE and the short-read sequencing data. The total and minor allele copy-number data in **a-h** are represented in black and blue, respectively. DEL, deletion-like rearrangement; DUP, duplication-like rearrangement; h2hINV, head-to-head inversion; t2tINV, tail-to-tail inversion. Lines with a square at the top represent single breakends, and lines with arrowheads mark insertions.

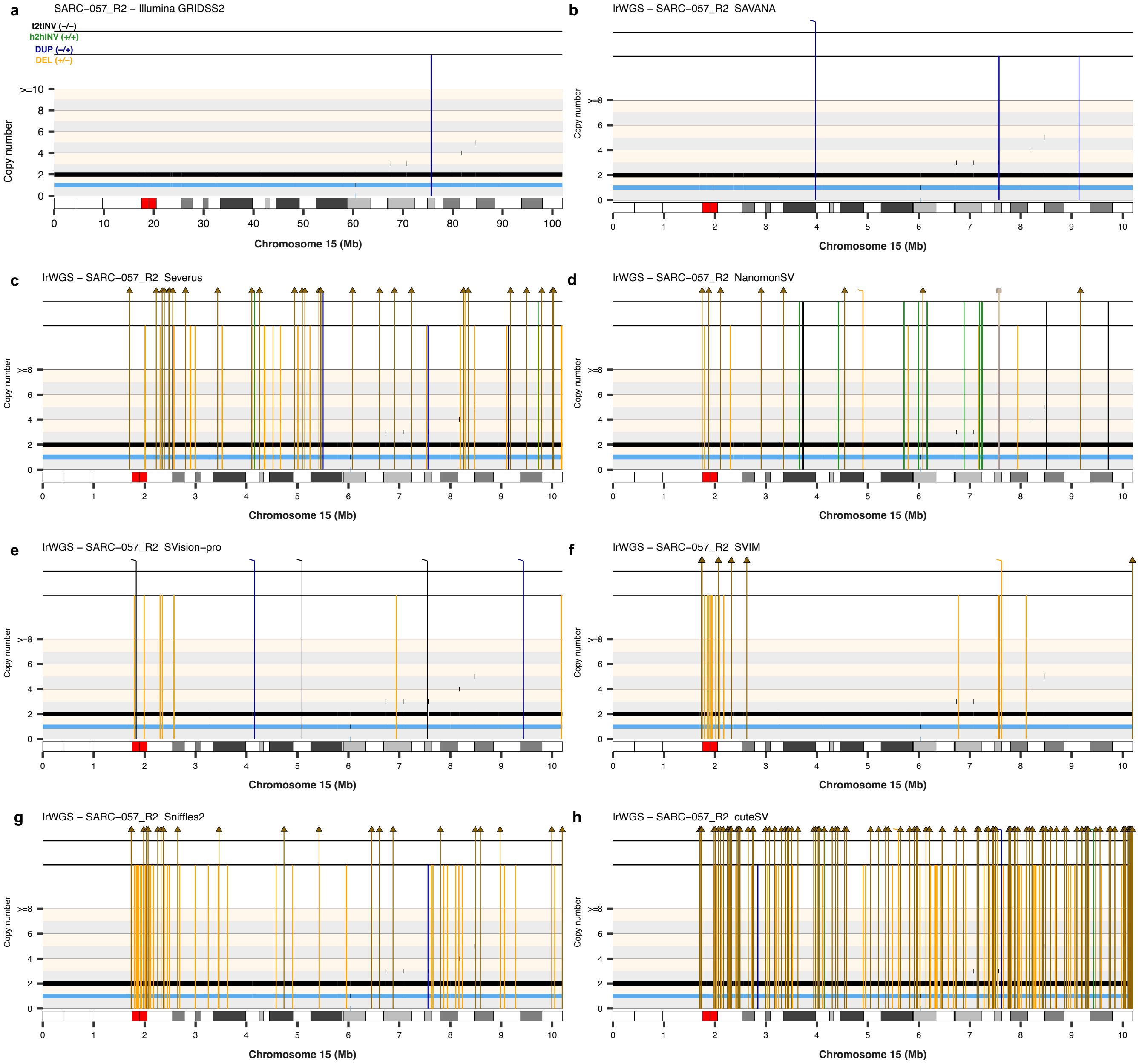

Supplementary Figure 15. Comparison of the SVs detected in tumour region 2 from the MPNST SARC-057.
(**a**) Somatic SVs and copy number profiles detected using GRIDSS2 and PURPLE in whole-genome short-read sequencing data. Somatic SVs detected in matched long-read nanopore whole-genome sequencing data (lrWGS) using SAVANA (**b**), Severus (**c**), NanomonSV (**d**), SVision-pro (**e**), SVIM (**f**), Sniffles2 (**g**) and cuteSV (**h**). The copy number profiles shown in **a**-**h** were calculated using PURPLE and the short-read sequencing data. The total and minor allele copy-number data in **a**-**h** are represented in black and blue, respectively. DEL, deletion-like rearrangement; DUP, duplication-like rearrangement; h2hINV, head-to-head inversion; t2tINV, tail-to-tail inversion. Lines with a square at the top represent single breakends, and lines with arrowheads mark insertions.

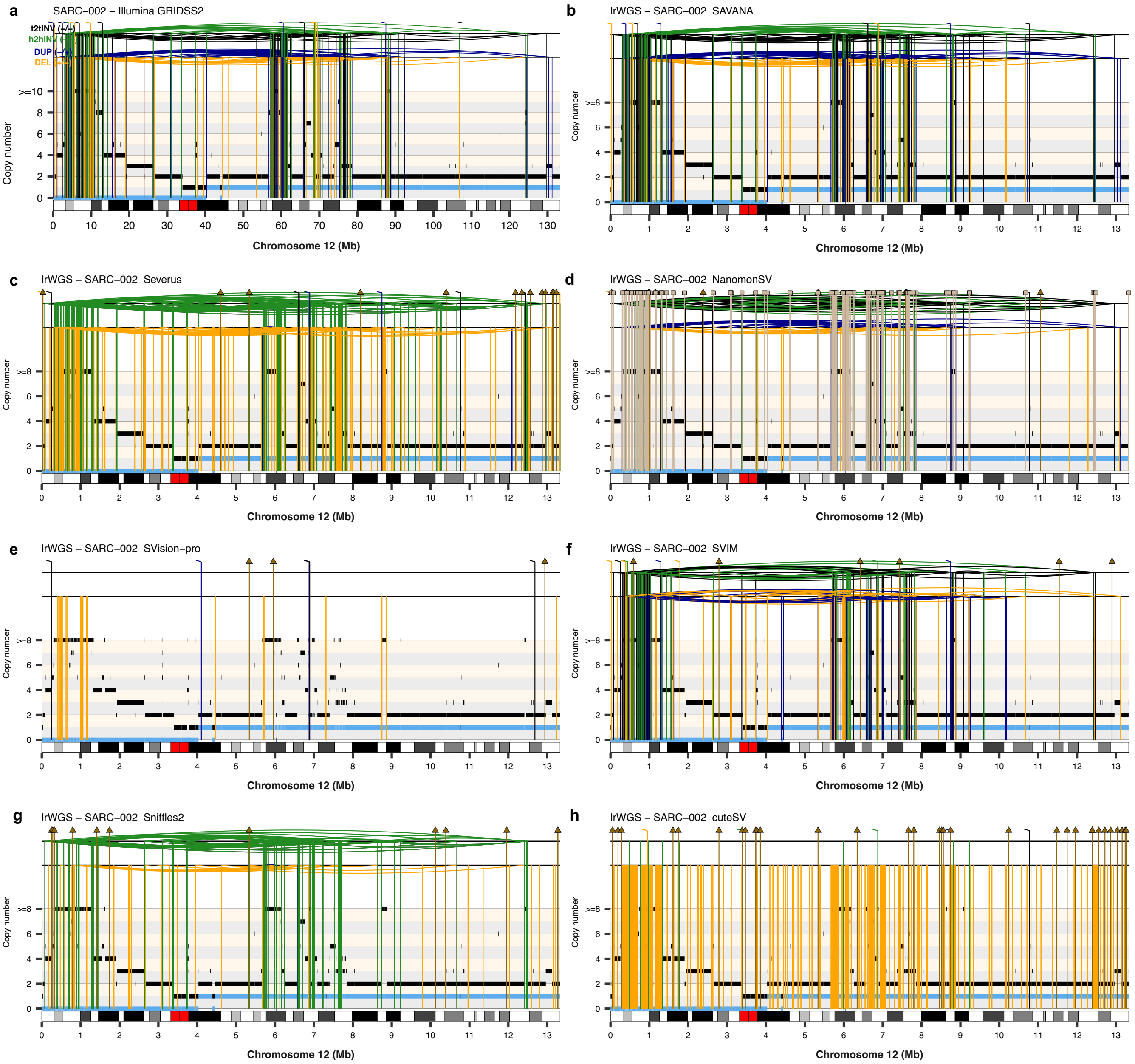

Supplementary Figure 16. Comparison of the SVs detected in the osteosarcoma SARC-002.
(**a**) Somatic SVs and copy number profiles detected using GRIDSS2 and PURPLE in whole-genome short-read sequencing data. Somatic SVs detected in matched long-read nanopore whole-genome sequencing data (lrWGS) using SAVANA (**b**), Severus (**c**), NanomonSV (**d**), SVision-pro (**e**), SVIM (**f**), Sniffles2 (**g**) and cuteSV (**h**). The copy number profiles shown in **a-h** were calculated using PURPLE and the short-read sequencing data. The total and minor allele copy-number data in **a-h** are represented in black and blue, respectively. DEL, deletion-like rearrangement; DUP, duplication-like rearrangement; h2hINV, head-to-head inversion; t2tINV, tail-to-tail inversion. Lines with a square at the top represent single breakends, and lines with arrowheads mark insertions.

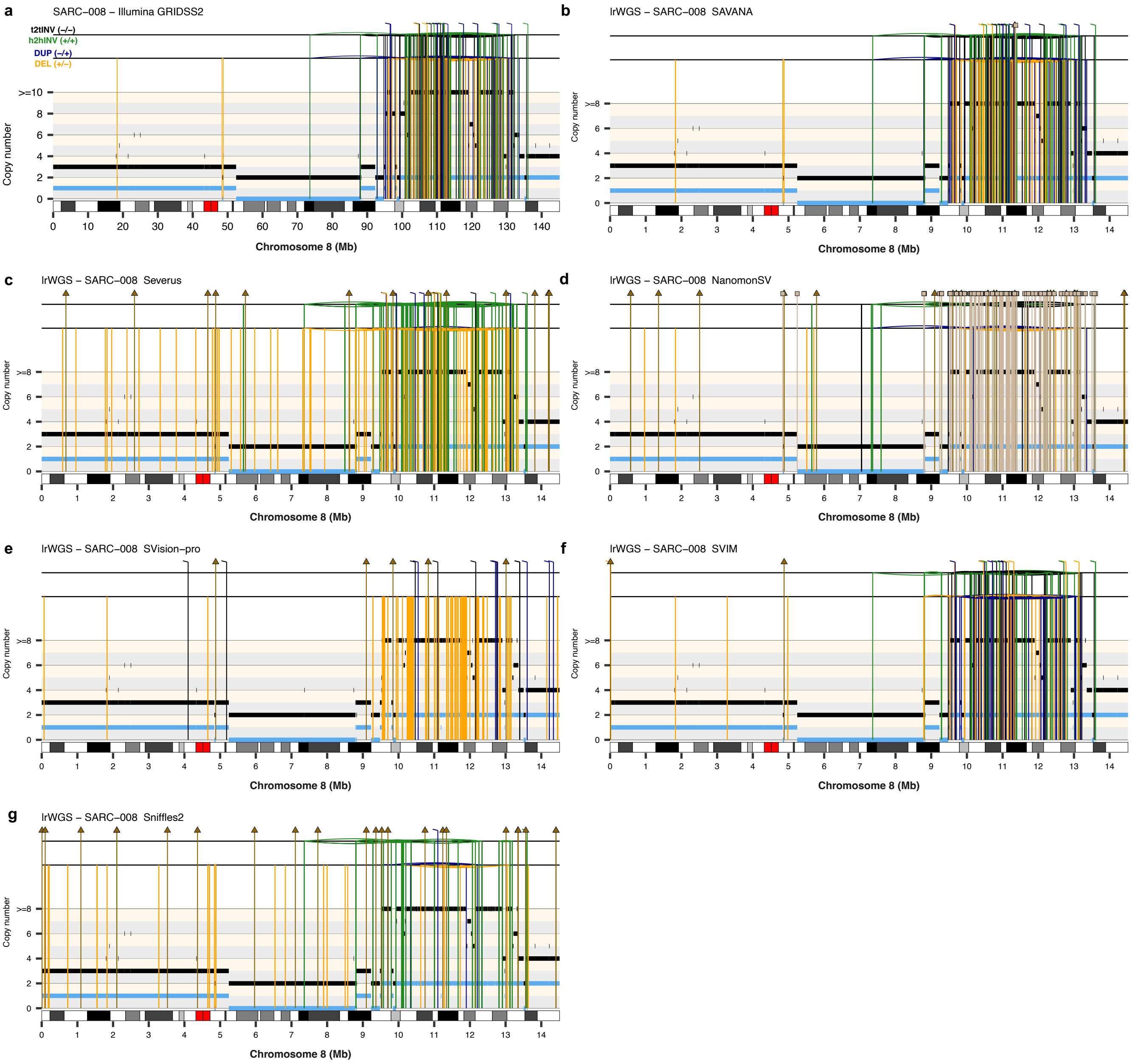

Supplementary Figure 17. Comparison of the SVs detected in the osteosarcoma SARC-008.
(**a**) Somatic SVs and copy number profiles detected using GRIDSS2 and PURPLE in whole-genome short-read sequencing data. Somatic SVs detected in matched long-read nanopore whole-genome sequencing data (lrWGS) using SAVANA (**b**), Severus (**c**), NanomonSV (**d**), SVision-pro (**e**), SVIM (**f**) and Sniffles2 (**g**). The copy number profiles shown in **a-h** were calculated using PURPLE and the short-read sequencing data. The total and minor allele copy-number data in **a-h** are represented in black and blue, respectively. DEL, deletion-like rearrangement; DUP, duplication-like rearrangement; h2hINV, head-to-head inversion; t2tINV, tail-to-tail inversion. Lines with a square at the top represent single breakends, and lines with arrowheads mark insertions.

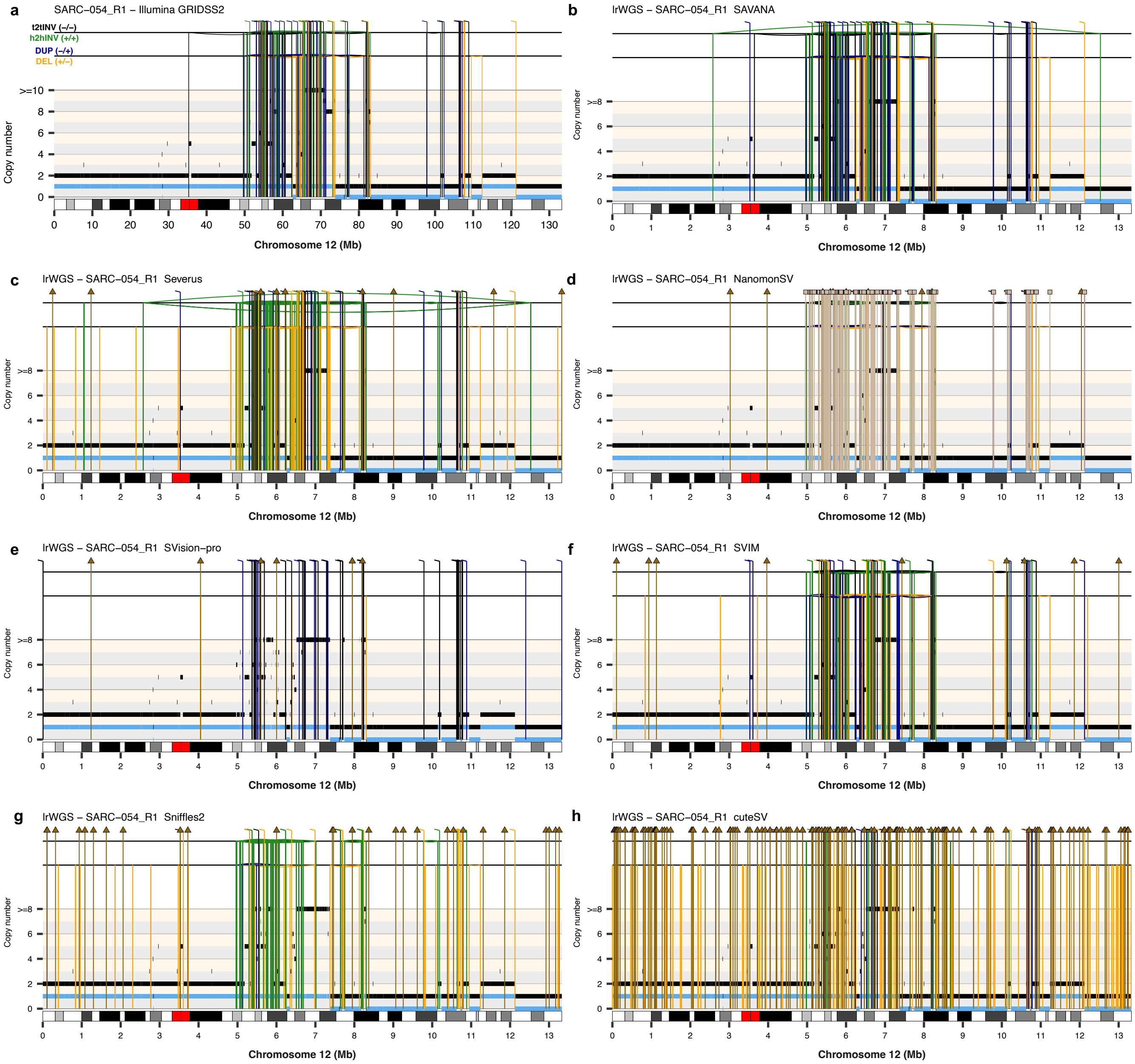

Supplementary Figure 18. Comparison of the SVs detected in the myxofibrosarcoma SARC-054.
(**a**) Somatic SVs and copy number profiles detected using GRIDSS2 and PURPLE in whole-genome short-read sequencing data. Somatic SVs detected in matched long-read nanopore whole-genome sequencing data (lrWGS) using SAVANA (**b**), Severus (**c**), NanomonSV (**d**), SVision-pro (**e**), SVIM (**f**), Sniffles2 (**g**) and cuteSV (**h**). The copy number profiles shown in **a-h** were calculated using PURPLE and the short-read sequencing data. The total and minor allele copy-number data in **a-h** are represented in black and blue, respectively. DEL, deletion-like rearrangement; DUP, duplication-like rearrangement; h2hINV, head-to-head inversion; t2tINV, tail-to-tail inversion. Lines with a square at the top represent single breakends, and lines with arrowheads mark insertions.

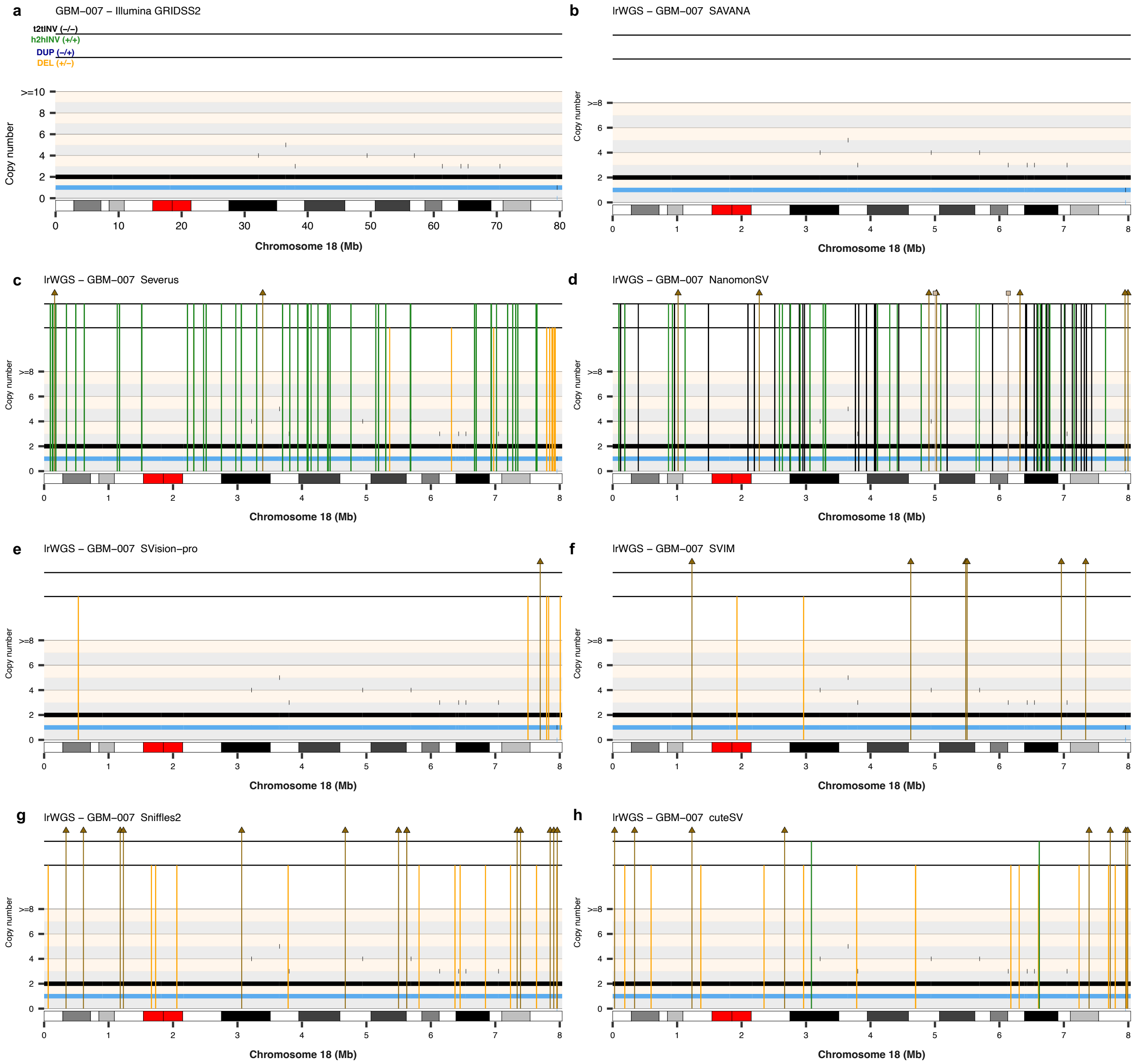

Supplementary Figure 19. Comparison of the SVs detected in the glioblastoma GBM-007.
(**a**) Somatic SVs and copy number profiles detected using GRIDSS2 and PURPLE in whole-genome short-read sequencing data. Somatic SVs detected in matched long-read nanopore whole-genome sequencing data (lrWGS) using SAVANA (**b**), Severus (**c**), NanomonSV (**d**), SVision-pro (**e**), SVIM (**f**), Sniffles2 (**g**) and cuteSV (**h**). The copy number profiles shown in **a-h** were calculated using PURPLE and the short-read sequencing data. The total and minor allele copy-number data in **a-h** are represented in black and blue, respectively. DEL, deletion-like rearrangement; DUP, duplication-like rearrangement; h2hINV, head-to-head inversion; t2tINV, tail-to-tail inversion. Lines with a square at the top represent single breakends, and lines with arrowheads mark insertions.

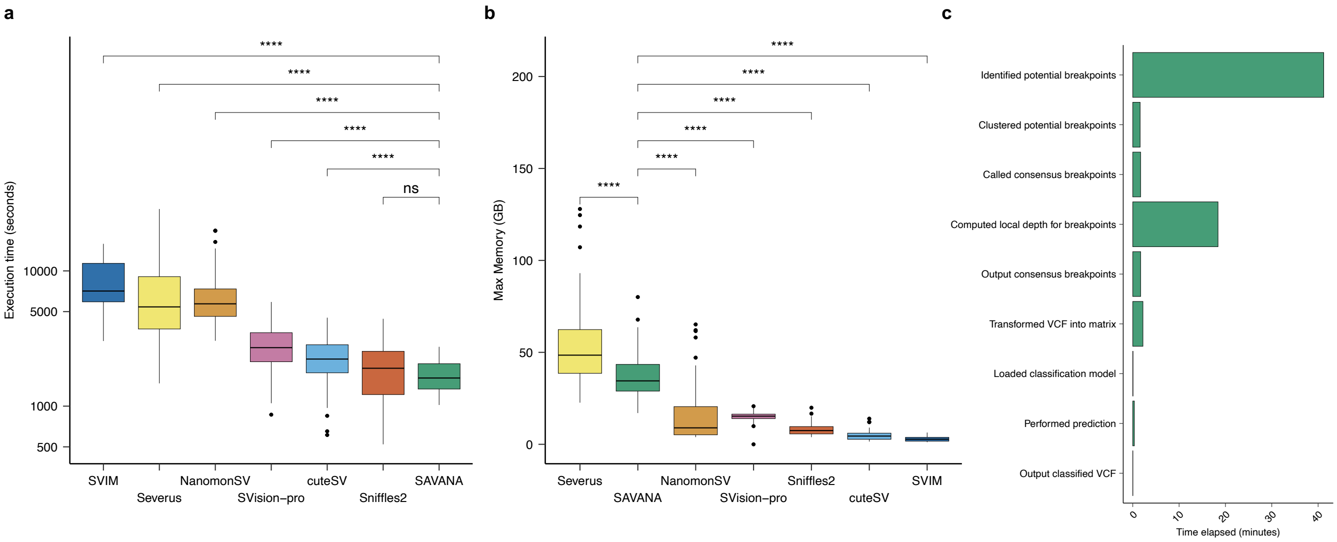

Supplementary Figure 20. Comparison of the execution time (a) and memory use (b) between SAVANA and existing SV detection algorithms across the cohort. (**c**) Running time for each step of SAVANA. Significance in **a-b** was assessed using the two-sided Wilcoxon's rank test (***P* < 0.001; *****P* < 0.00001). To assess the computational performance of algorithms, each caller was run on the cohort of split tumour BAMs on a SLURM scheduler, where 16 CPUs were made available for multiprocessing (if applicable). Callers that allowed threads to be set via command-line argument (SAVANA, Severus, NanomonSV, Sniffles2, and cuteSV) were set using the relevant thread argument. Each caller was assessed on total execution time and maximum memory.

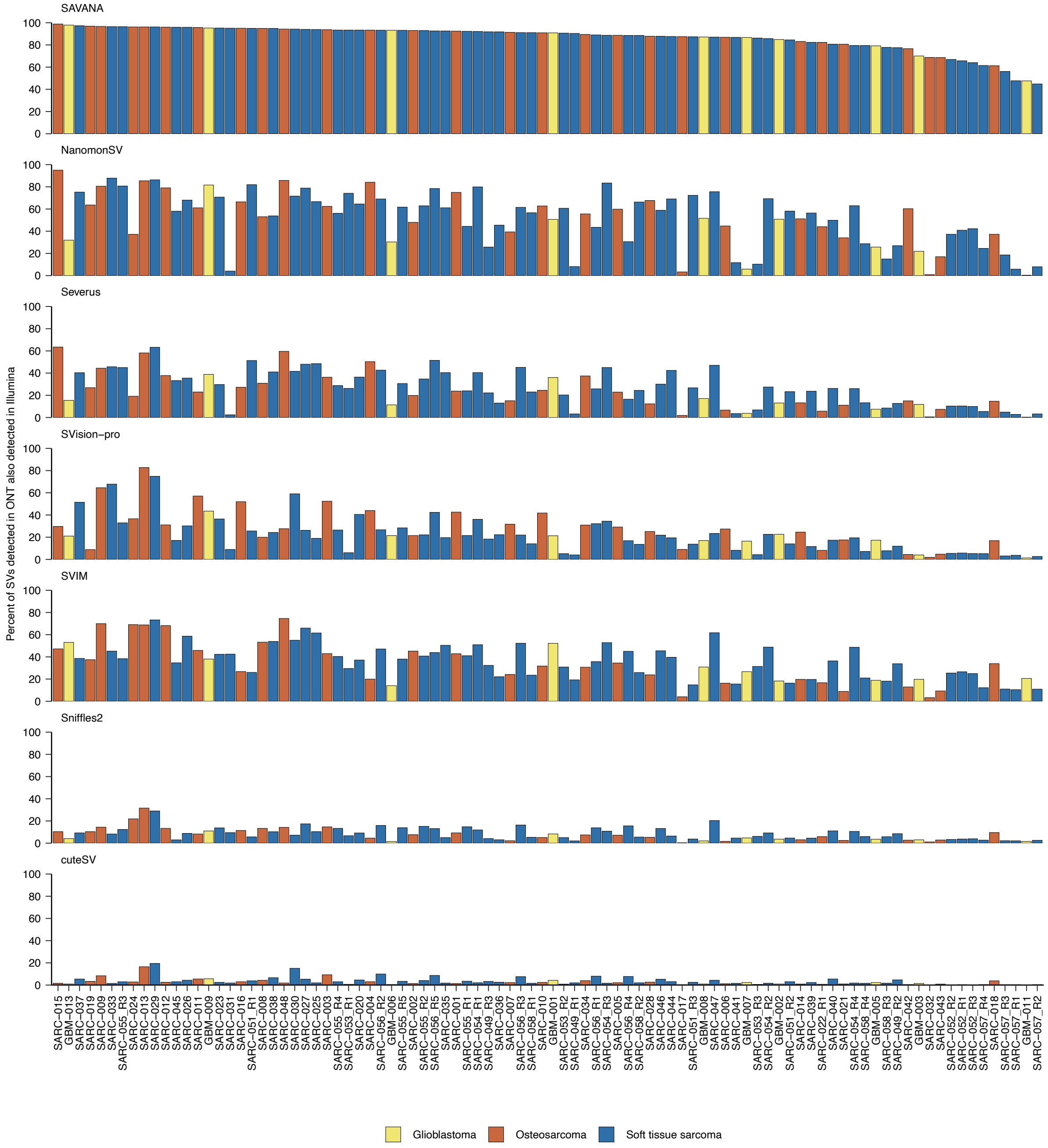

Supplementary Figure 21. Comparison between short and long read sequencing data for somatic SV detection. Each bar reports the percent of somatic SVs detected in nanopore long-read WGS data that are also detected in matched Illumina WGS data.

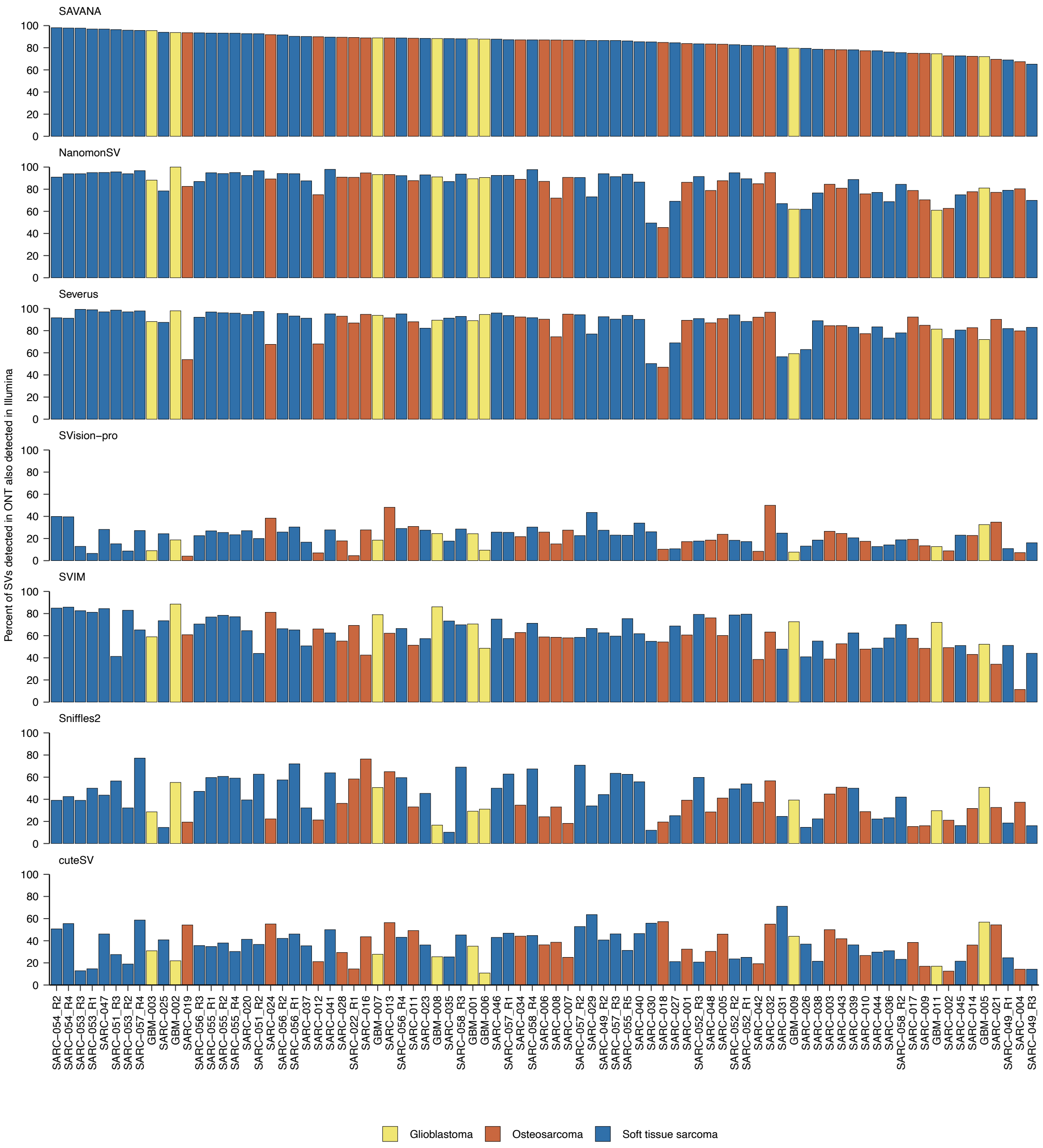

Supplementary Figure 22. Comparison between short and long read sequencing data for somatic SV detection. Each bar reports the percent of somatic SVs detected in Illumina WGS data that are also detected in matched nanopore long-read WGS data.

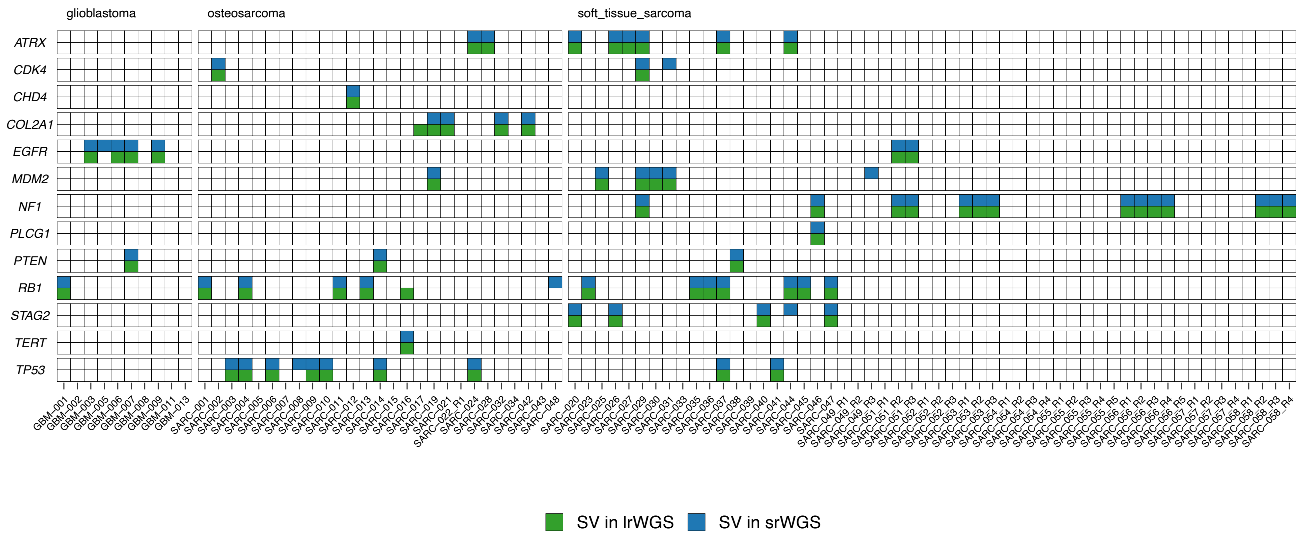

Supplementary Figure 23. Comparison of short-read WGS (srWGS; blue) and long-read WGS (lrWGS; green) for the detection of SVs in cancer driver genes. Cancer driver genes in each tumour were identified through the analysis of the Illumina data using GRIDSS and PURPLE. SVs in long-read data were detected using SAVANA. Overall, srWGS and lrWGS perform on par for the detection of SVs in cancer driver genes.

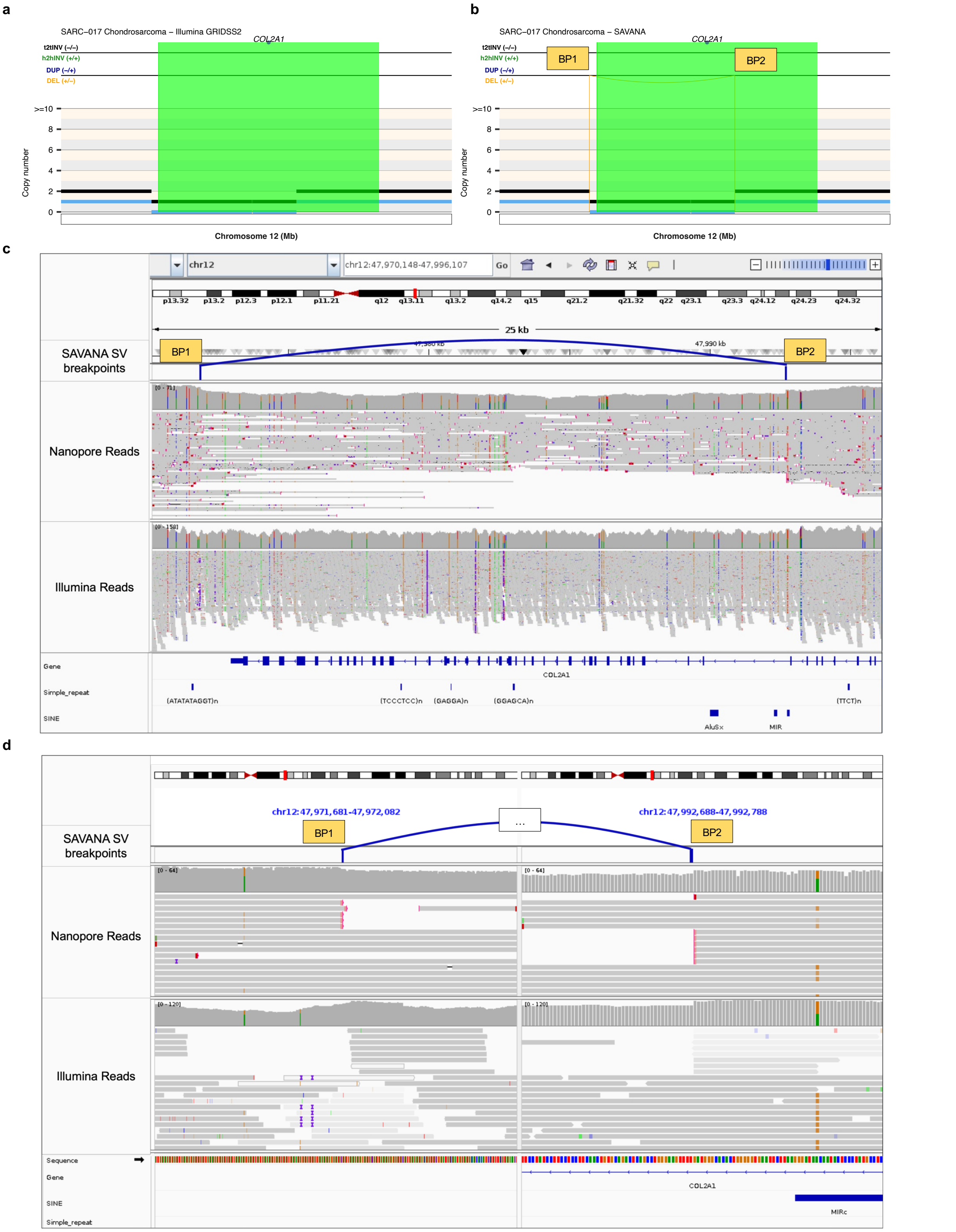

Supplementary Figure 24. Example of an SV disrupting *COL2A1* in a central conventional chondrosarcoma missed by short-read sequencing. (**a**) Somatic SVs and SCNAs detected using GRIDSS2 and PURPLE in short-read whole-genome sequencing data. (**b**) Somatic SVs and SCNAs detected in matched long-read nanopore whole-genome sequencing data using SAVANA. The total and minor allele copy-number data in **a-b** are represented in black and blue, respectively. DEL, deletion-like rearrangement; DUP, duplication-like rearrangement; h2hINV, head-to-head inversion; t2tINV, tail-to-tail inversion. The gene *COL2A1* is highlighted in green showing its overlap with the second breakpoint (BP) of a deletion-like SV identified by SAVANA (**c**) The top and bottom panels show the sequencing reads from nanopore and Illumina sequencing of the same tumour, respectively, in the regions of the genome corresponding to the SV breakpoints (marked by BP1 and BP2) and SCNA changepoints. (**d**) The same sample showing Nanopore and Illumina reads, zoomed to show read alignment to the breakpoints of the SV. The quality of the alignment of the Illumina reads is poor, which results in poor mapping quality (light grey alignments). As a result, the breakpoints cannot be reliably detected using Illumina sequencing.

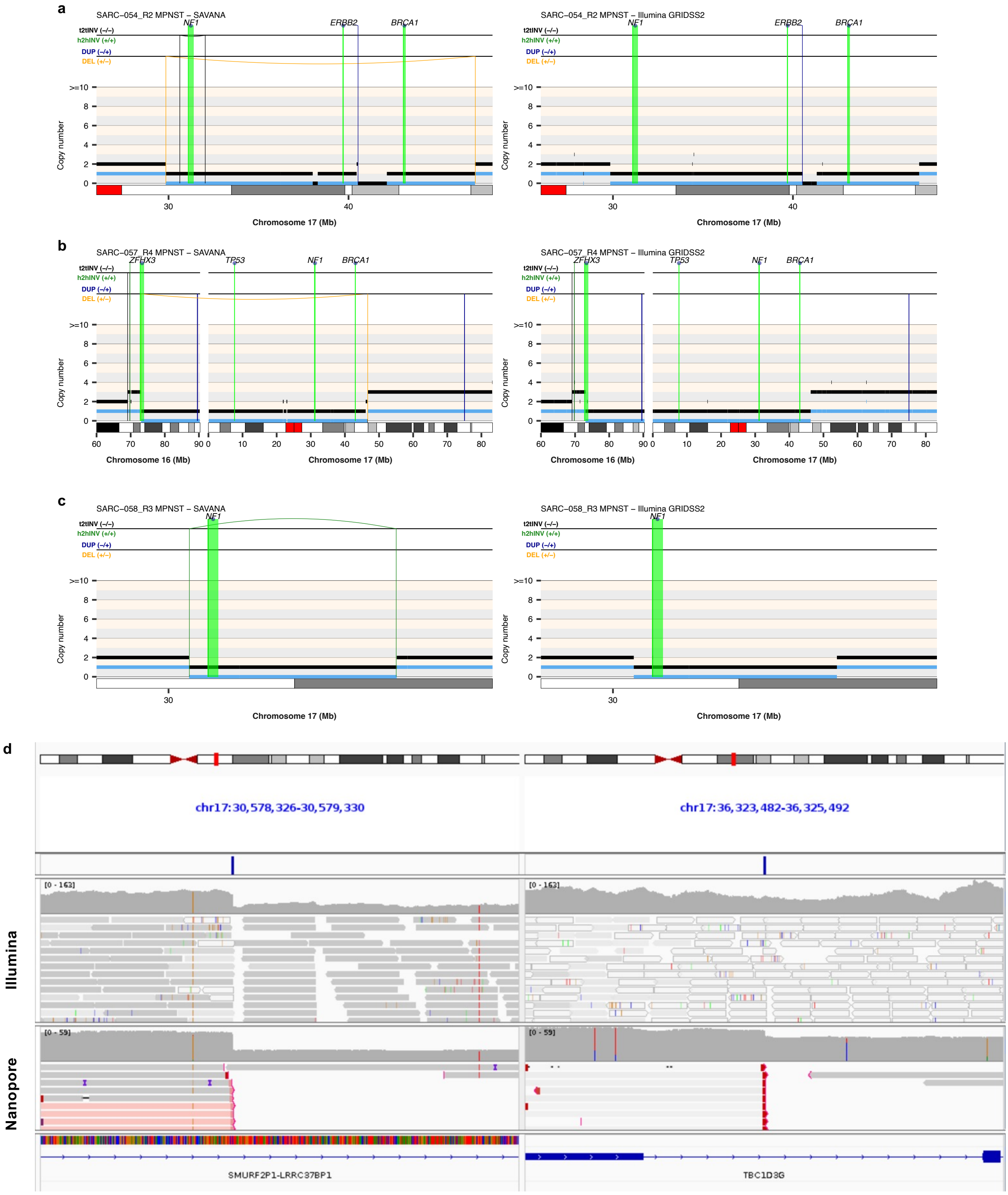

Supplementary Figure 25. Somatic SVs and SCNAs mapping to the *NF1* locus in malignant peripheral nerve sheath tumours (MPNSTs) SARC-054 (**a**), SARC-057 (**b**) and SARC-058 (**c**). The SVs and SCNAs detected using SAVANA and long reads are shown on the left, and the SVs and SCNAS detected using Illumina WGS and GRIDSS/PURPLE are shown on the right. These three examples illustrate two cases in which the rearrangements that lead to the loss of *NF1* (the main driver of MPNST development) are only detected by SAVANA using the long-read data. The total and minor allele copy-number data are represented in black and blue, respectively. DEL, deletion-like rearrangement; DUP, duplication-like rearrangement; h2hINV, head-to-head inversion; t2tINV, tail-to-tail inversion. (**d**) Illumina and nanopore read alignments for the SV shown in panel **c.** The breakpoints can only be reliably reconstructed using the long reads. The short reads show low mapping quality, as indicated by the light colour of sequencing reads.

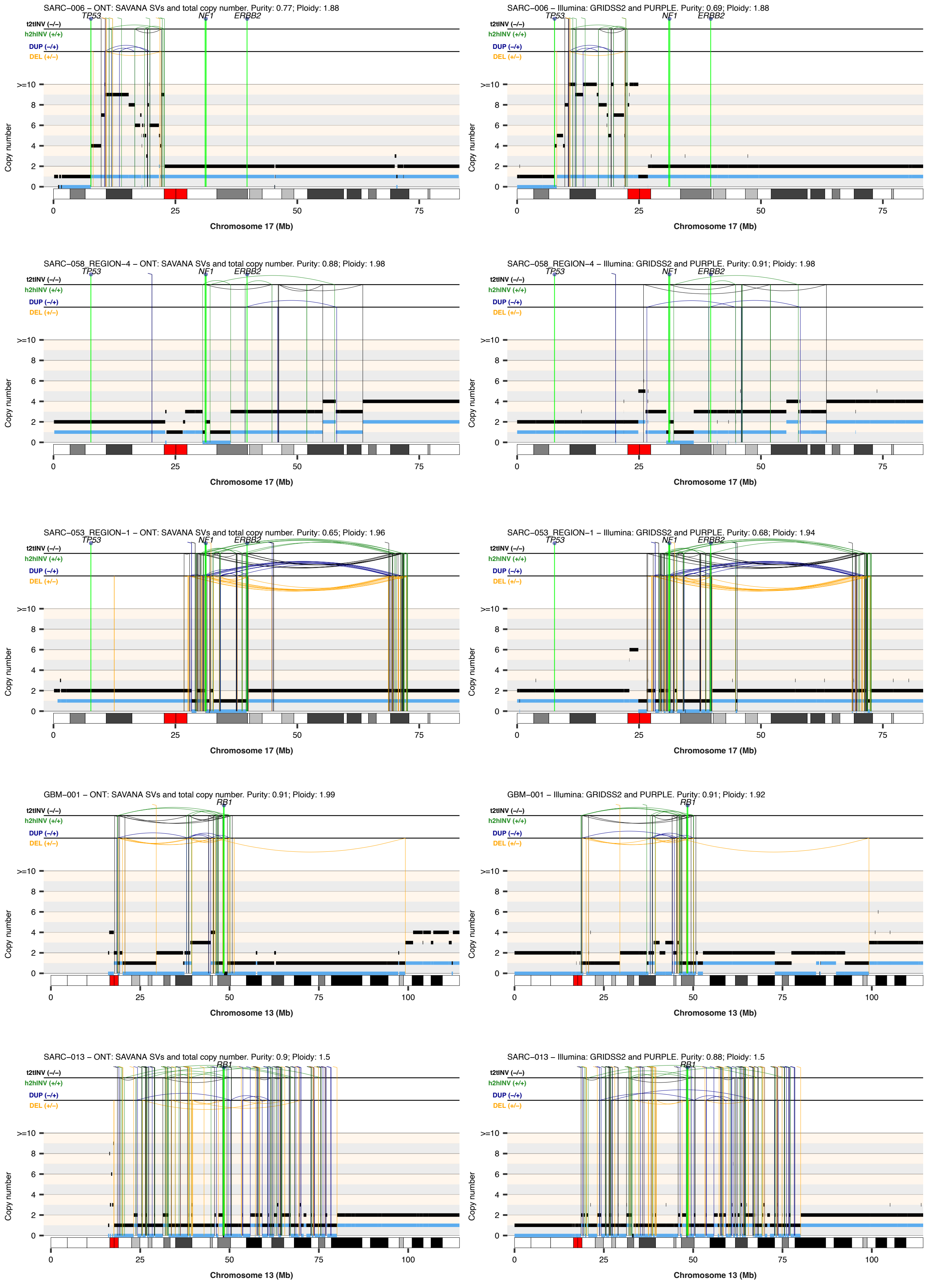

Supplementary Figure 26. Somatic rearrangement and copy number profiles calculated using short-read data analyzed with GRIDSS and PURPLE against long-read data analyzed using SAVANA.
(**Left**) Somatic SVs and SCNAs detected in matched long-read nanopore whole-genome sequencing data using SAVANA. (**Right**) Somatic SVs and copy number profiles detected using GRIDSS2 and PURPLE in whole-genome short-read sequencing data. The total and minor allele copy-number data are represented in black and blue, respectively. DEL, deletion-like rearrangement; DUP, duplication-like rearrangement; h2hINV, head-to-head inversion; t2tINV, tail-to-tail inversion. Lines with arrowheads mark insertions.

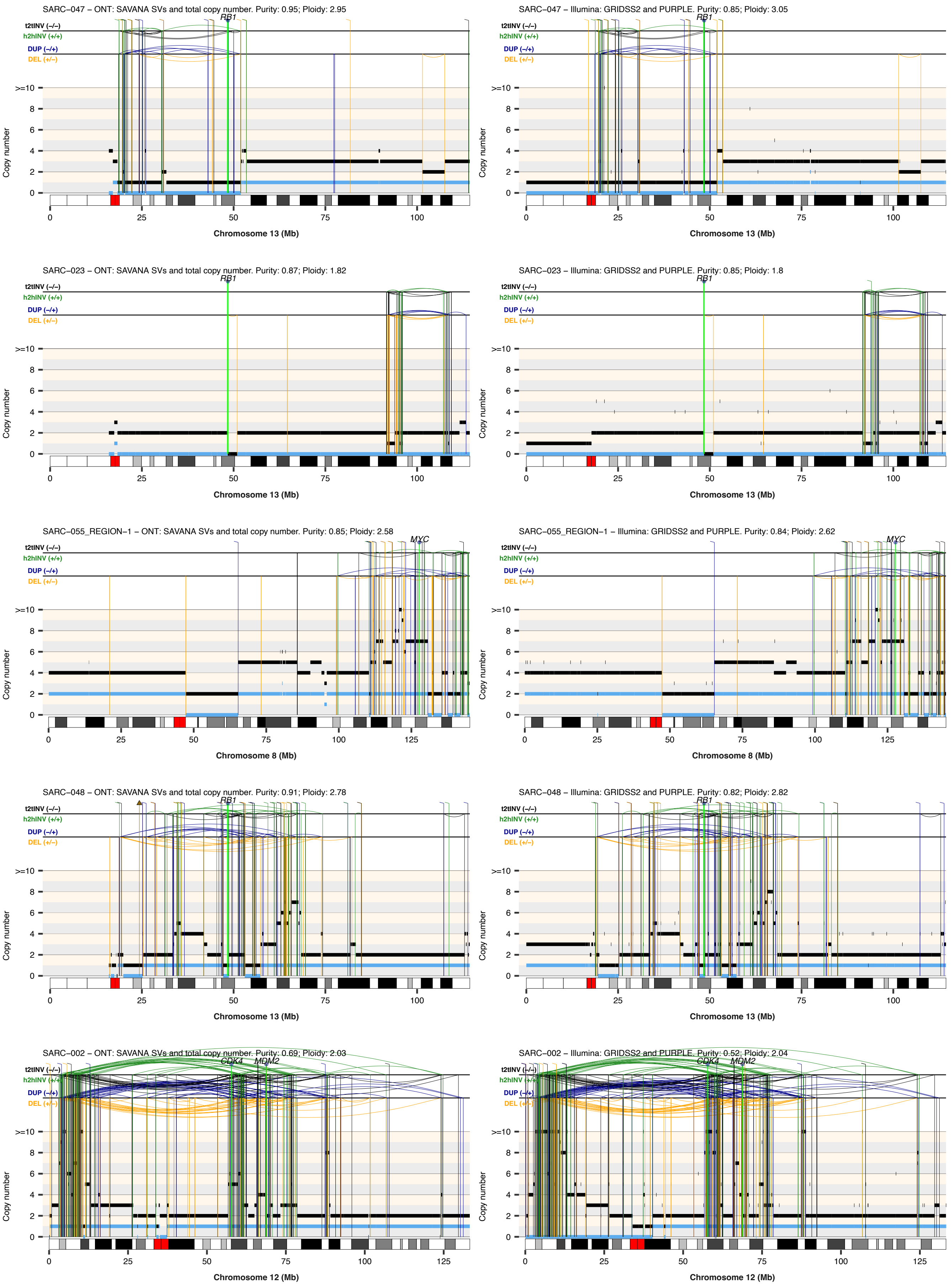

Supplementary Figure 27. Somatic rearrangement and copy number profiles calculated using short-read data analyzed with GRIDSS and PURPLE against long-read data analyzed using SAVANA. (**Left**) Somatic SVs and SCNAs detected in matched long-read nanopore whole-genome sequencing data using SAVANA. (**Right**) Somatic SVs and copy number profiles detected using GRIDSS2 and PURPLE in whole-genome short-read sequencing data. The total and minor allele copy-number data are represented in black and blue, respectively. DEL, deletion-like rearrangement; DUP, duplication-like rearrangement; h2hINV, head-to-head inversion; t2tINV, tail-to-tail inversion. Lines with arrowheads mark insertions.

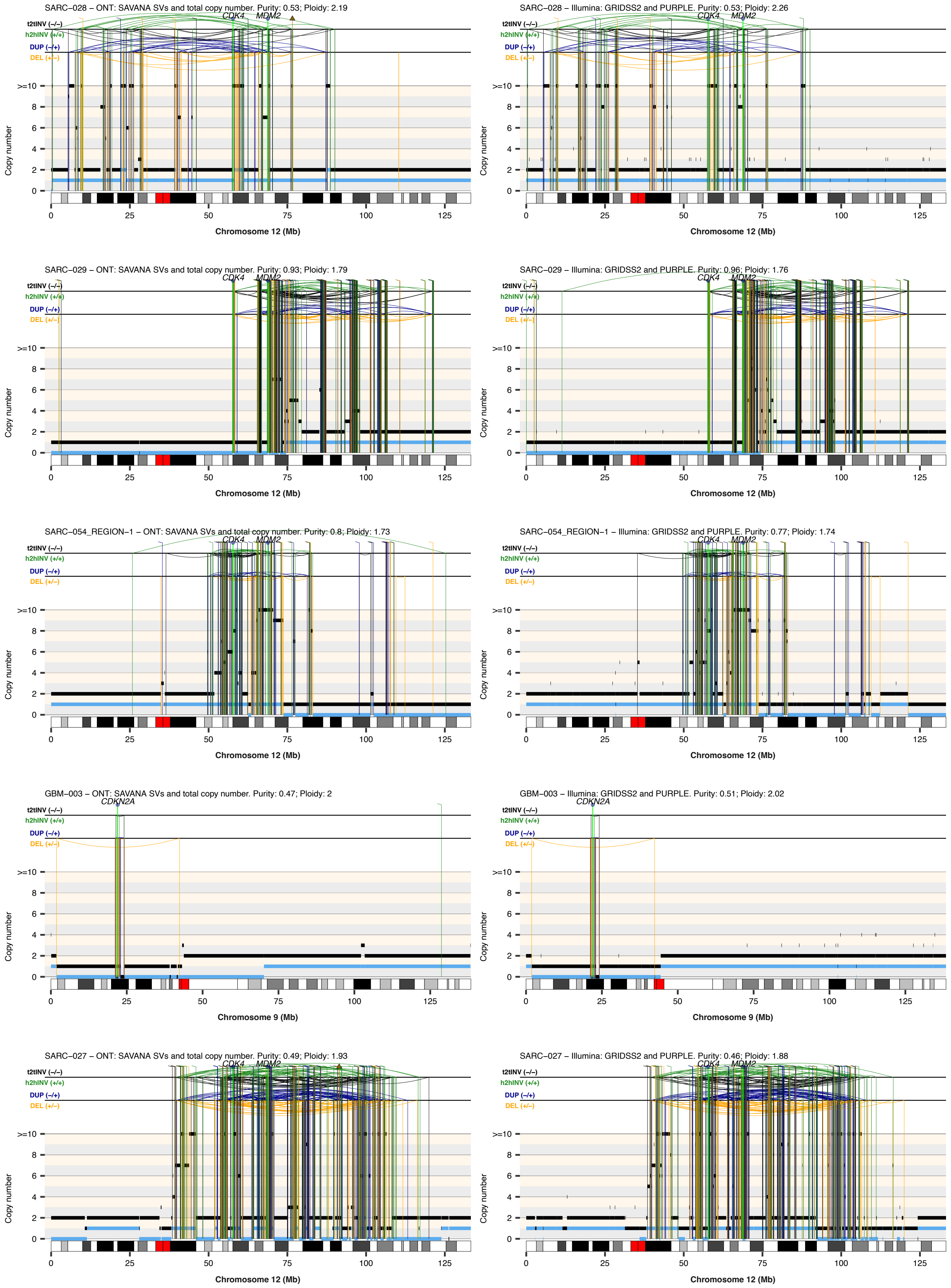

Supplementary Figure 28. Somatic rearrangement and copy number profiles calculated using short-read data analyzed with GRIDSS and PURPLE against long-read data analyzed using SAVANA. (**Left**) Somatic SVs and SCNAs detected in matched long-read nanopore whole-genome sequencing data using SAVANA. (**Right**) Somatic SVs and copy number profiles detected using GRIDSS2 and PURPLE in whole-genome short-read sequencing data. The total and minor allele copy-number data are represented in black and blue, respectively. DEL, deletion-like rearrangement; DUP, duplication-like rearrangement; h2hINV, head-to-head inversion; t2tINV, tail-to-tail inversion. Lines with arrowheads mark insertions.

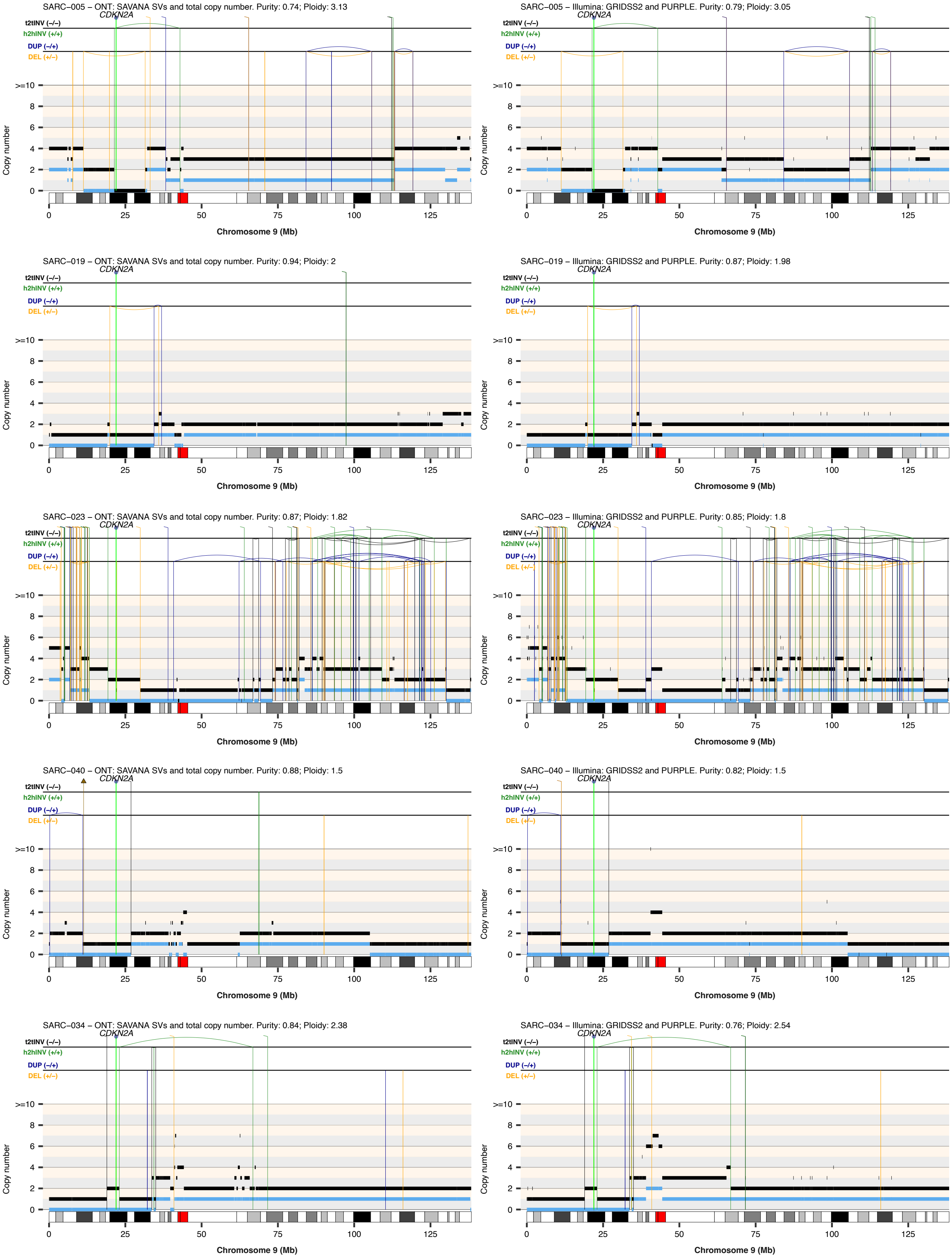

Supplementary Figure 29. Somatic rearrangement and copy number profiles calculated using short-read data analyzed with GRIDSS and PURPLE against long-read data analyzed using SAVANA. (**Left**) Somatic SVs and SCNAs detected in matched long-read nanopore whole-genome sequencing data using SAVANA. (**Right**) Somatic SVs and copy number profiles detected using GRIDSS2 and PURPLE in whole-genome short-read sequencing data. The total and minor allele copy-number data are represented in black and blue, respectively. DEL, deletion-like rearrangement; DUP, duplication-like rearrangement; h2hINV, head-to-head inversion; t2tINV, tail-to-tail inversion. Lines with arrowheads mark insertions.

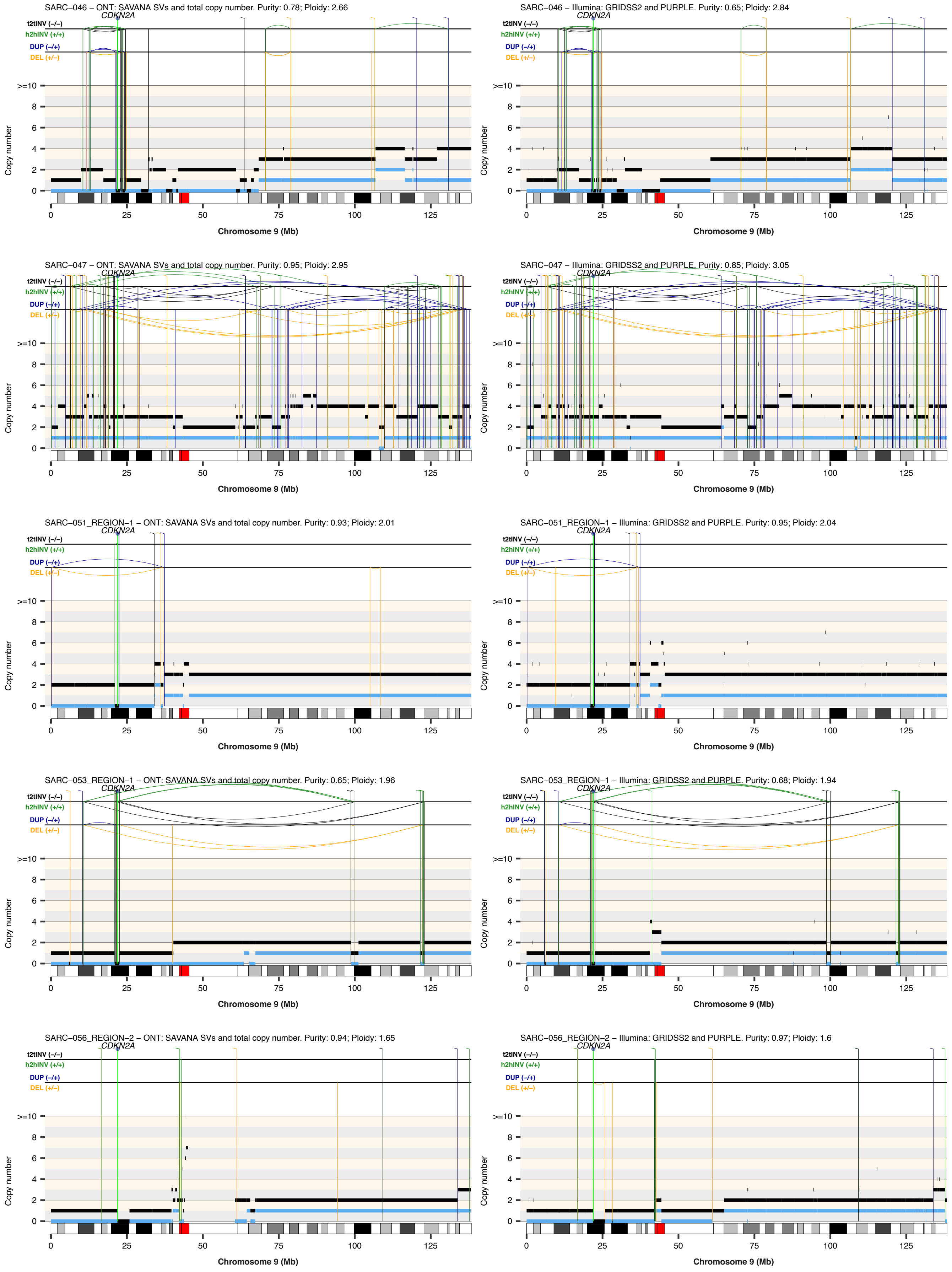

Supplementary Figure 30. Somatic rearrangement and copy number profiles calculated using short-read data analyzed with GRIDSS and PURPLE against long-read data analyzed using SAVANA. (**Left**) Somatic SVs and SCNAs detected in matched long-read nanopore whole-genome sequencing data using SAVANA. (**Right**) Somatic SVs and copy number profiles detected using GRIDSS2 and PURPLE in whole-genome short-read sequencing data. The total and minor allele copy-number data are represented in black and blue, respectively. DEL, deletion-like rearrangement; DUP, duplication-like rearrangement; h2hINV, head-to-head inversion; t2tINV, tail-to-tail inversion. Lines with a square at the top represent single breakends, and lines with arrowheads mark insertions.

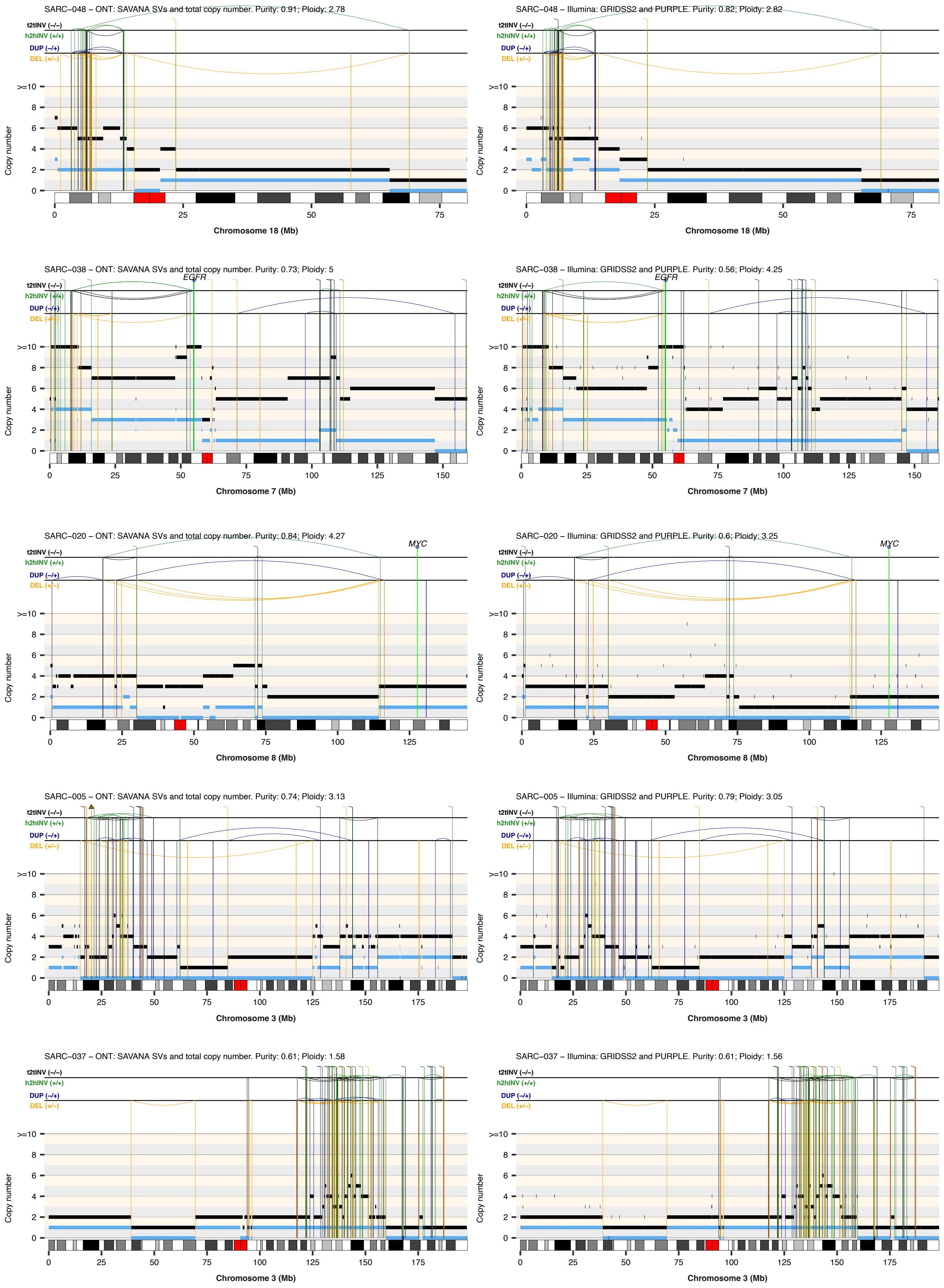

Supplementary Figure 31. Somatic rearrangement and copy number profiles calculated using short-read data analyzed with GRIDSS and PURPLE against long-read data analyzed using SAVANA. (**Left**) Somatic SVs and SCNAs detected in matched long-read nanopore whole-genome sequencing data using SAVANA. (**Right**) Somatic SVs and copy number profiles detected using GRIDSS2 and PURPLE in whole-genome short-read sequencing data. The total and minor allele copy-number data are represented in black and blue, respectively. DEL, deletion-like rearrangement; DUP, duplication-like rearrangement; h2hINV, head-to-head inversion; t2tINV, tail-to-tail inversion. Lines with a square at the top represent single breakends, and lines with arrowheads mark insertions.

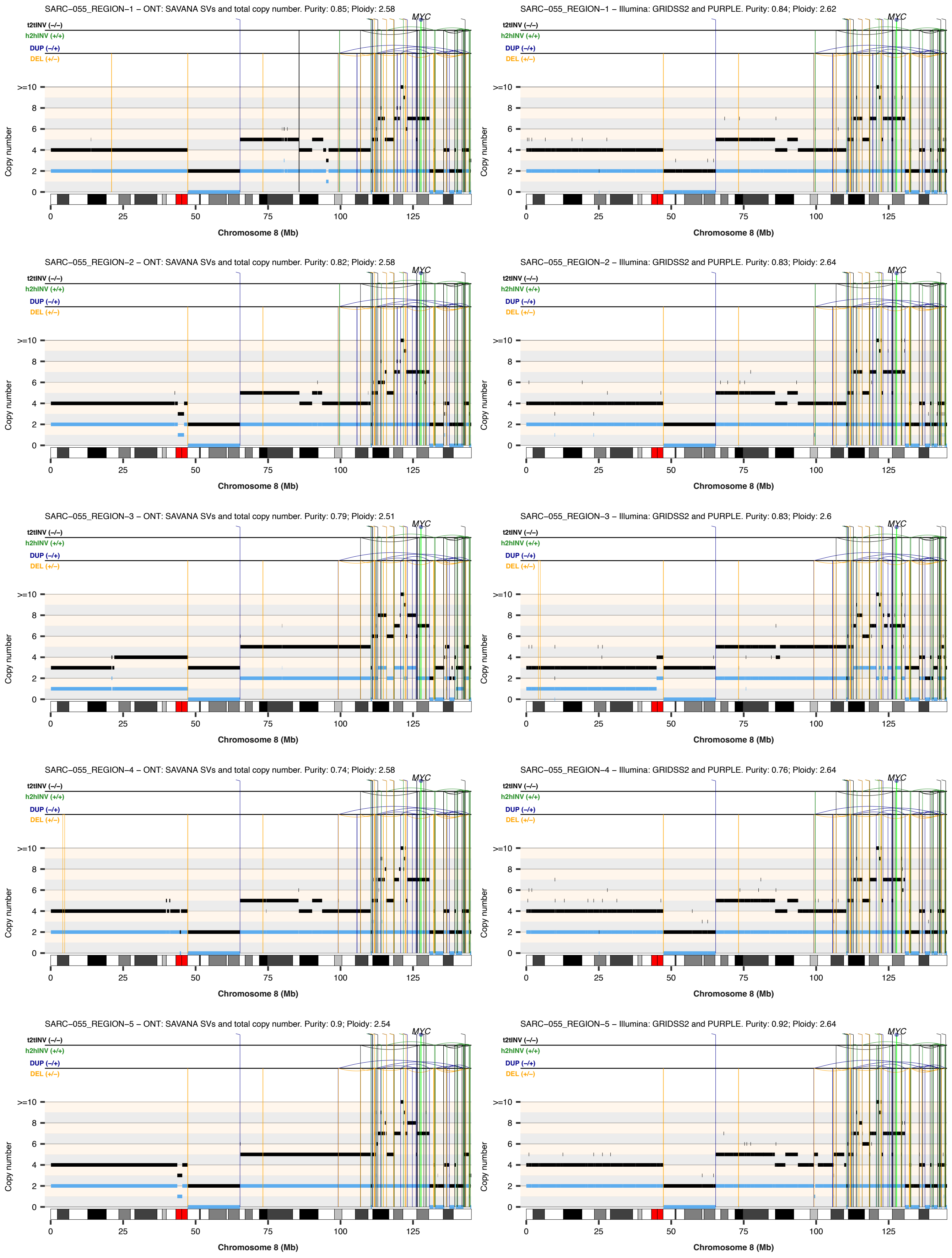

Supplementary Figure 32. Somatic rearrangement and copy number profiles calculated using short-read data analyzed with GRIDSS and PURPLE against long-read data analyzed using SAVANA. (**Left**) Somatic SVs and SCNAs detected in matched long-read nanopore whole-genome sequencing data using SAVANA. (**Right**) Somatic SVs and copy number profiles detected using GRIDSS2 and PURPLE in whole-genome short-read sequencing data. The total and minor allele copy-number data are represented in black and blue, respectively. DEL, deletion-like rearrangement; DUP, duplication-like rearrangement; h2hINV, head-to-head inversion; t2tINV, tail-to-tail inversion. Lines with arrowheads mark insertions.

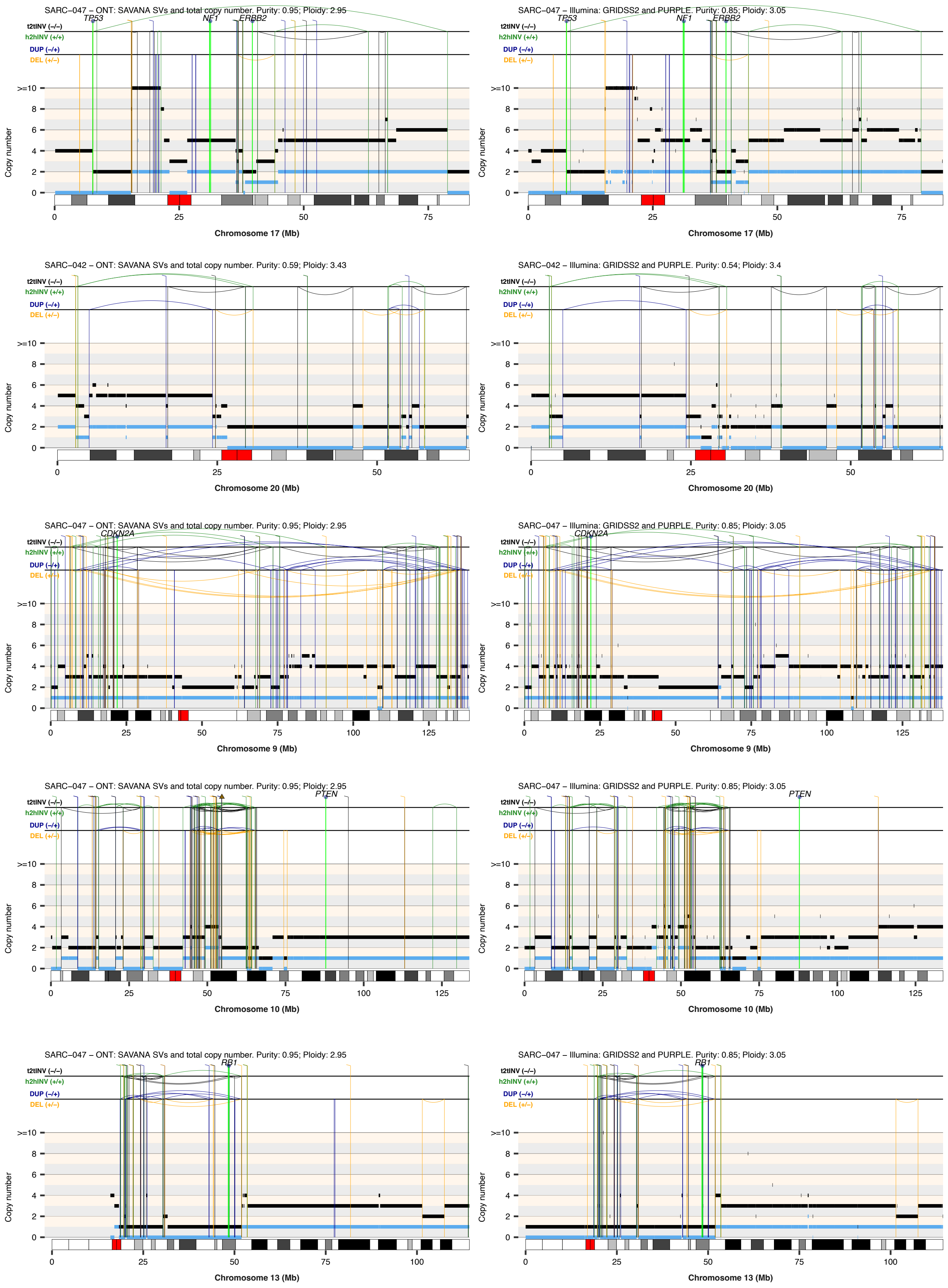

Supplementary Figure 33. Somatic rearrangement and copy number profiles calculated using short-read data analyzed with GRIDSS and PURPLE against long-read data analyzed using SAVANA.(**Left**) Somatic SVs and SCNAs detected in matched long-read nanopore whole-genome sequencing data using SAVANA. (**Right**) Somatic SVs and copy number profiles detected using GRIDSS2 and PURPLE in whole-genome short-read sequencing data. The total and minor allele copy-number data are represented in black and blue, respectively. DEL, deletion-like rearrangement; DUP, duplication-like rearrangement; h2hINV, head-to-head inversion; t2tINV, tail-to-tail inversion. Lines with a square at the top represent single breakends, and lines with arrowheads mark insertions.

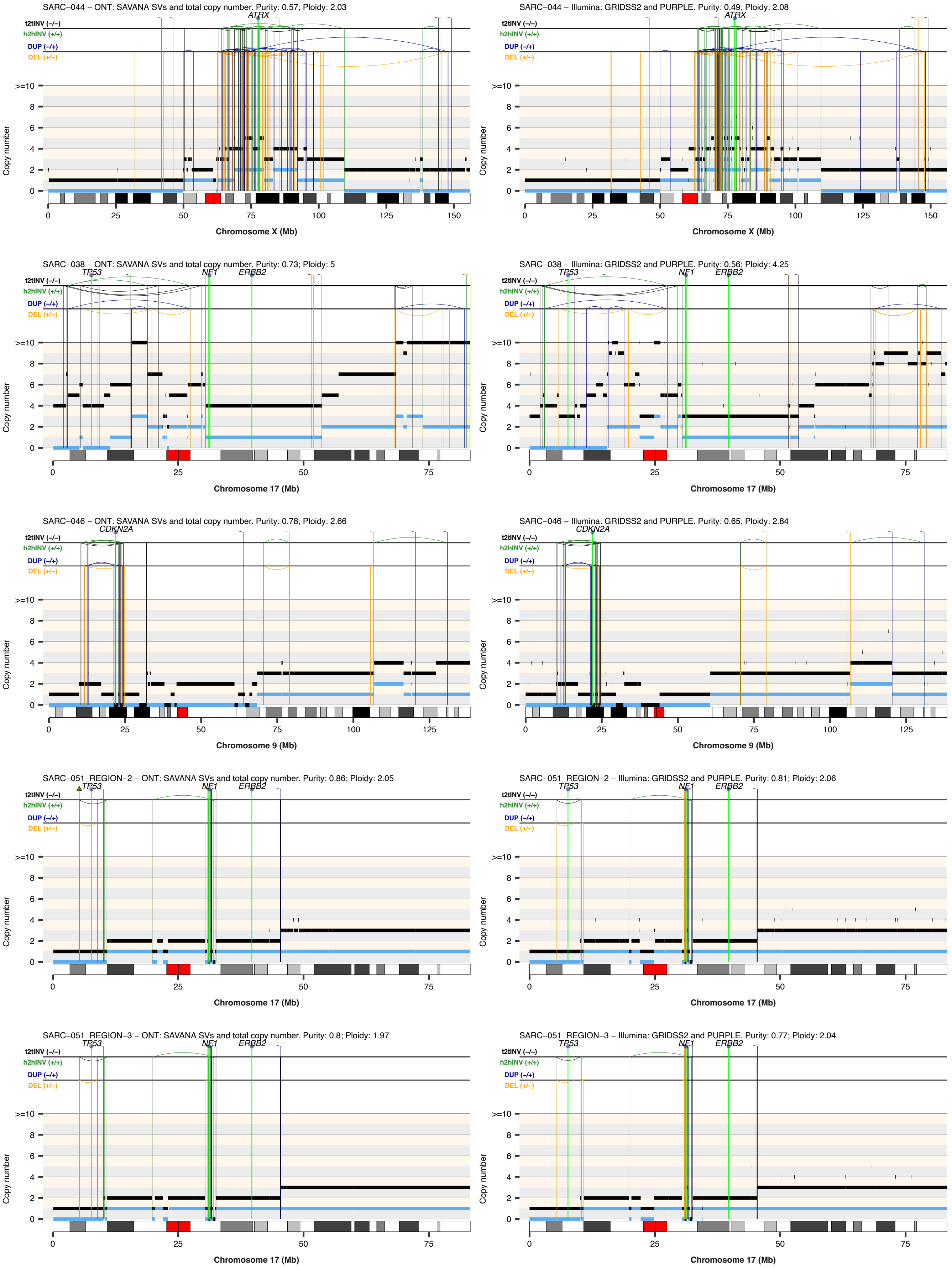

Supplementary Figure 34. Somatic rearrangement and copy number profiles calculated using short-read data analyzed with GRIDSS and PURPLE against long-read data analyzed using SAVANA. (**Left**) Somatic SVs and SCNAs detected in matched long-read nanopore whole-genome sequencing data using SAVANA. (**Right**) Somatic SVs and copy number profiles detected using GRIDSS2 and PURPLE in whole-genome short-read sequencing data. The total and minor allele copy-number data are represented in black and blue, respectively. DEL, deletion-like rearrangement; DUP, duplication-like rearrangement; h2hINV, head-to-head inversion; t2tINV, tail-to-tail inversion. Lines with arrowheads mark insertions.

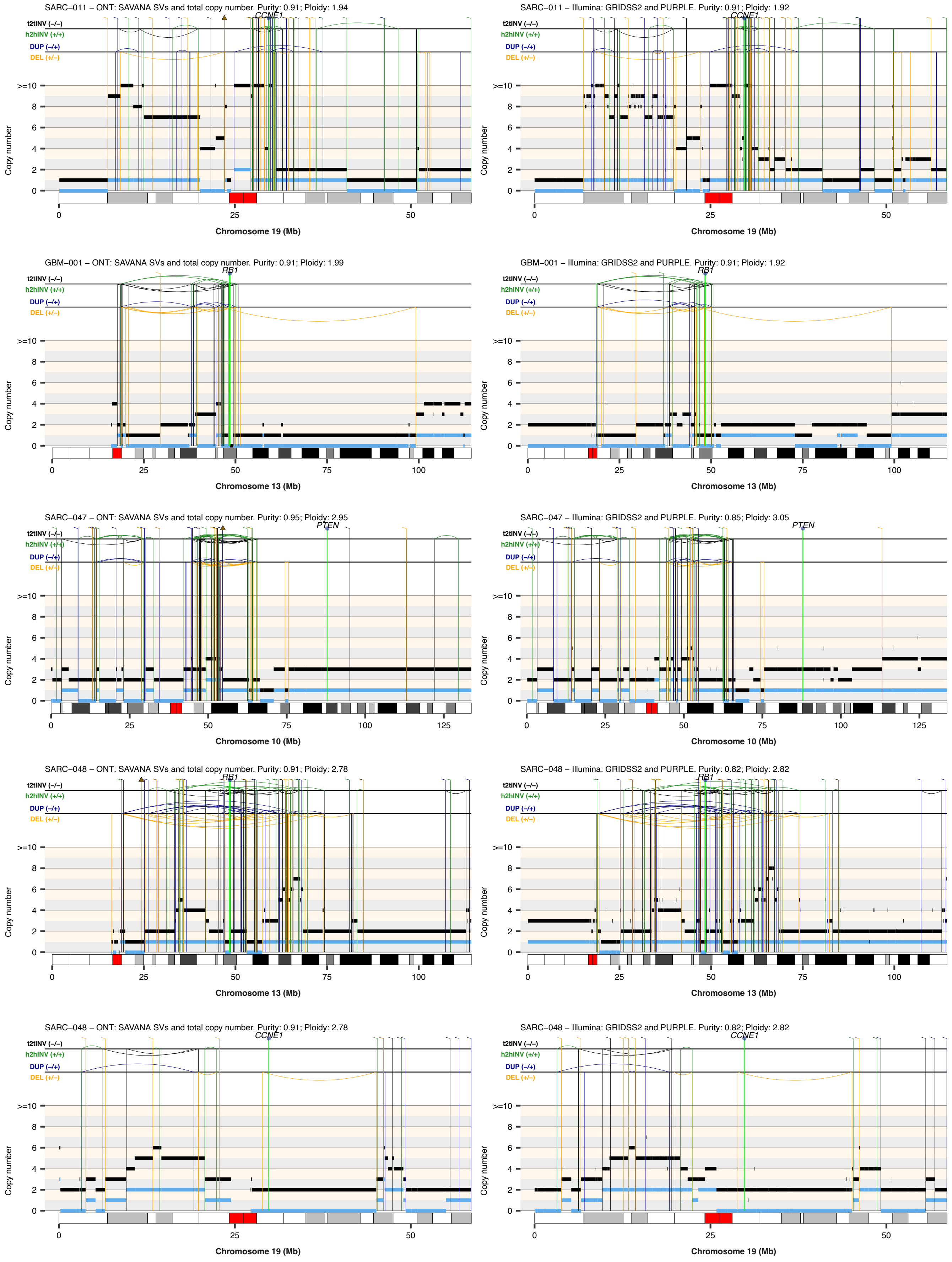

Supplementary Figure 35. Somatic rearrangement and copy number profiles calculated using short-read data analyzed with GRIDSS and PURPLE against long-read data analyzed using SAVANA. (**Left**) Somatic SVs and SCNAs detected in matched long-read nanopore whole-genome sequencing data using SAVANA. (**Right**) Somatic SVs and copy number profiles detected using GRIDSS2 and PURPLE in whole-genome short-read sequencing data. The total and minor allele copy-number data are represented in black and blue, respectively. DEL, deletion-like rearrangement; DUP, duplication-like rearrangement; h2hINV, head-to-head inversion; t2tINV, tail-to-tail inversion. Lines with arrowheads mark insertions.

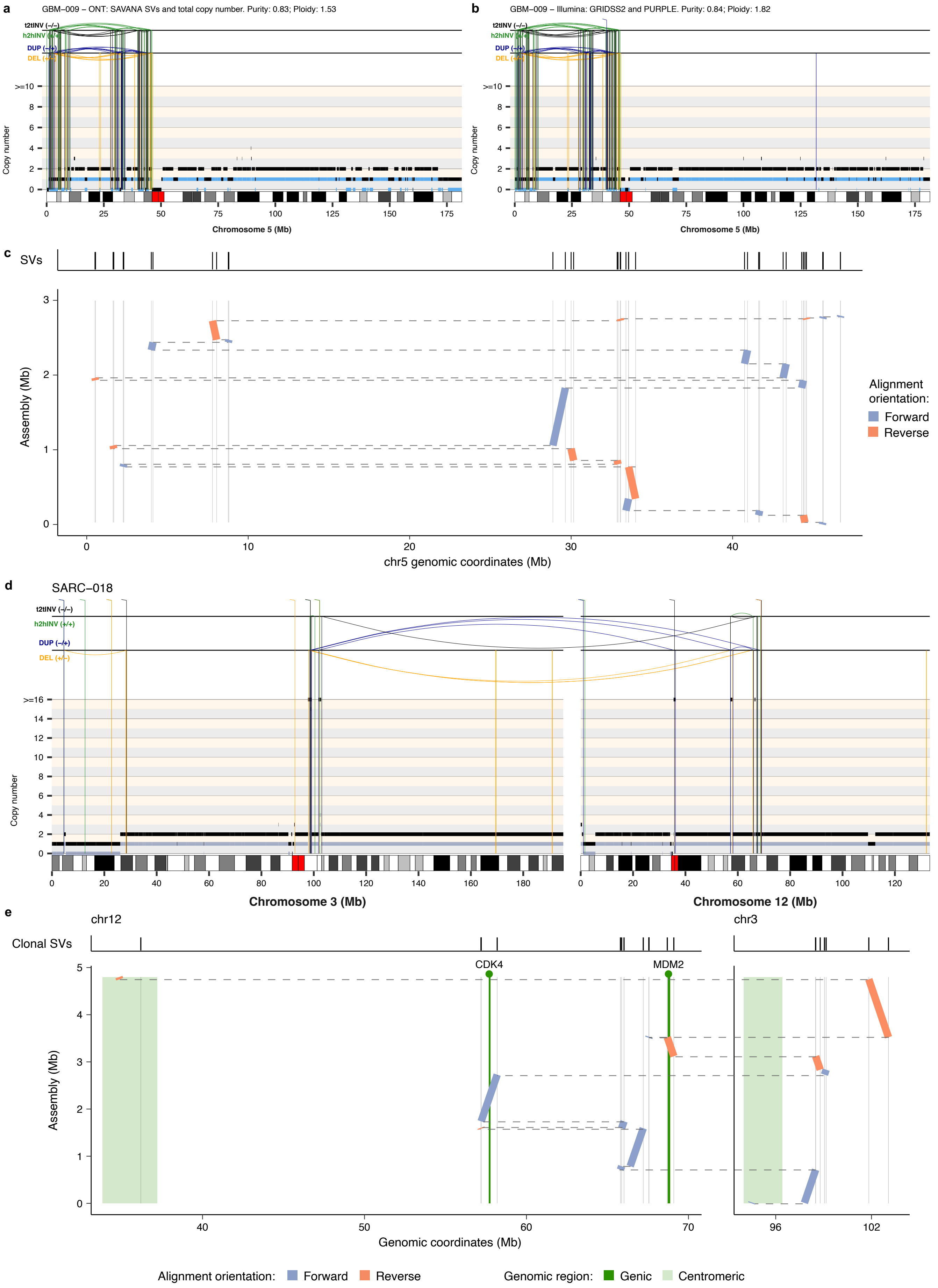

Supplementary Figure 36. Application of haplotype-resolved somatic SV analysis using SAVANA to the assembly of derivative chromosomes generated by complex genomic rearrangements.
Somatic SV and SCNA profile for chromosome 5 (**a-b**) involving a complex genomic rearrangement in tumour GBM-009. The total and minor allele copy-number data are represented in black and blue, respectively. DEL, deletion-like rearrangement; DUP, duplication-like rearrangement; h2hINV, head-to-head inversion; t2tINV, tail-to-tail inversion. Lines with a square at the top represent single breakends, and lines with arrowheads mark insertions. (**c**) Assembly of the complex genomic rearrangement shown in **a-b** using haplotype-resolved SVs detected by SAVANA. The plot illustrates the alignment of the assembly to chromosome 5 of the human reference genome, revealing multiple connected SVs across the chromosome. The vertical lines in the top panel represent the coordinates of the somatic breakpoints detected by SAVANA. **(d)** Somatic SV and somatic copy number profiles for chromosomes 3 and 12 for SARC-018. (**e**) The plot shows the alignment of the assembled contig for each parental allele (arbitrarily labelled as “allele 1” and “allele 2”) to chromosomes 3 and 12 of the human reference genome.

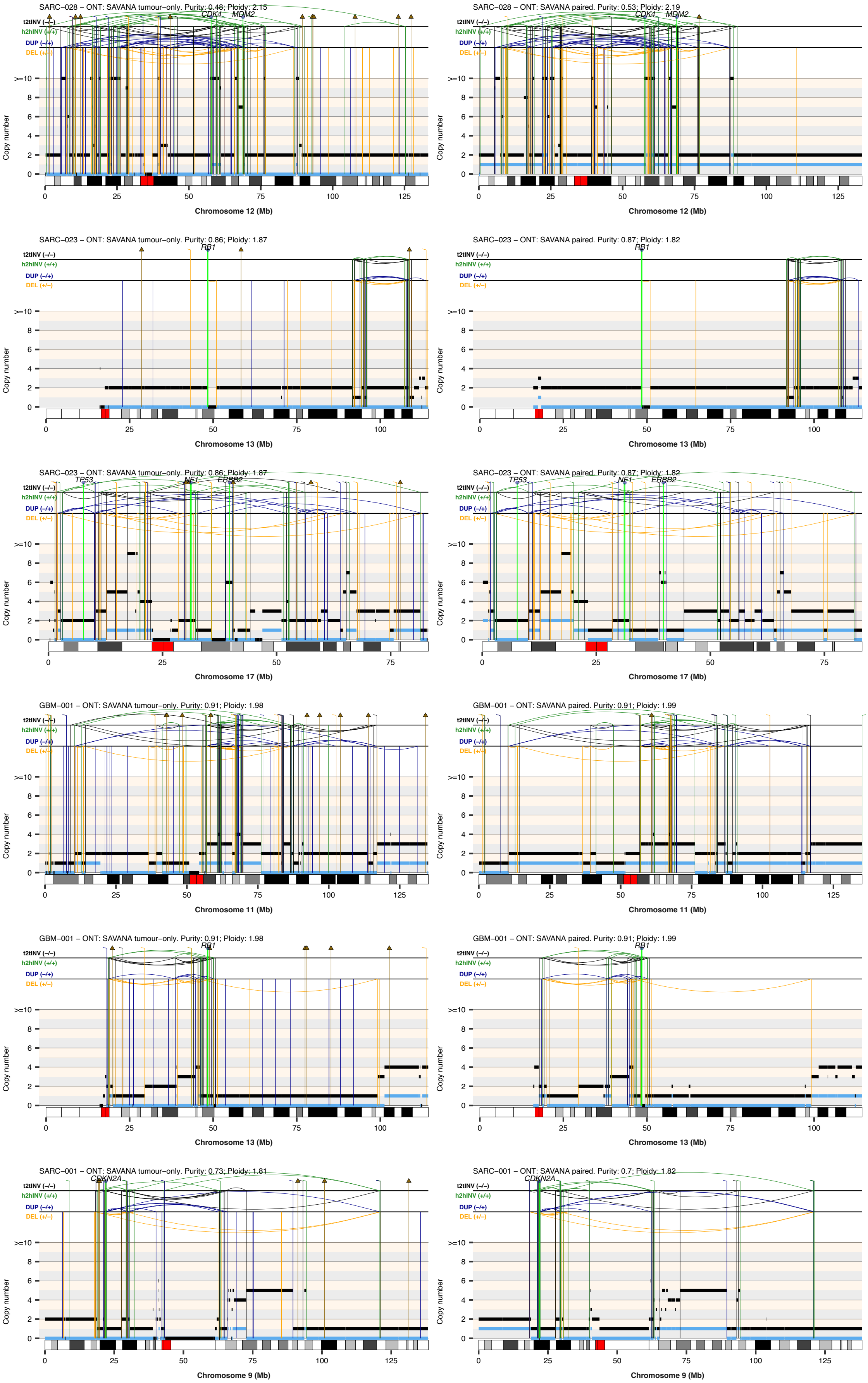

Supplementary Figure 37. Comparison between SAVANA run with a matched germline sample (paired mode) and without (tumour-only mode). The somatic SVs and SCNAs detected using SAVANA in tumour-only and paired mode are shown on the left and right, respectively. Overall, the SCNAs and SVs detected in either mode are highly correlated, and the SVs affecting cancer genes (e.g., *RB1, TP53, ERBB2* and *NF1*) and the associated changes in copy number (including LOH) are detected reliably in tumour-only mode. The total and minor allele copy-number data are represented in black and blue, respectively. DEL, deletion-like rearrangement; DUP, duplication-like rearrangement; h2hINV, head-to-head inversion; t2tINV, tail-to-tail inversion.
