# Supplementary material for: SAVANA: reliable analysis of somatic structural variants and copy number aberrations using long-read sequencing
Source: Nat Methods. 2025 May 28;22(7):1436–46. doi: 10.1038/s41592-025-02708-0 (PMC12240814; doi:10.1038/s41592-025-02708-0)
Supplement: Supplementary file 2 — Reporting Summary [file 41592_2025_2708_MOESM2_ESM.pdf]

nature portfolio

Corresponding author(s): Prof. Adrienne M. Flanagan
Dr. Isidro Cortes-Ciriano

Last updated by author(s): 04/03/2025

Reporting Summary

Nature Portfolio wishes to improve the reproducibility of the work that we publish. This form provides structure for consistency and transparency in reporting. For further information on Nature Portfolio policies, see our Editorial Policies and the Editorial Policy Checklist.

Statistics

For all statistical analyses, confirm that the following items are present in the figure legend, table legend, main text, or Methods section.

n/a	Confirmed	
☐	☒	The exact sample size (*n*) for each experimental group/condition, given as a discrete number and unit of measurement
☐	☒	A statement on whether measurements were taken from distinct samples or whether the same sample was measured repeatedly
☐	☒	The statistical test(s) used AND whether they are one- or two-sided<br>*Only common tests should be described solely by name; describe more complex techniques in the Methods section.*
☐	☒	A description of all covariates tested
☐	☒	A description of any assumptions or corrections, such as tests of normality and adjustment for multiple comparisons
☐	☒	A full description of the statistical parameters including central tendency (e.g. means) or other basic estimates (e.g. regression coefficient) AND variation (e.g. standard deviation) or associated estimates of uncertainty (e.g. confidence intervals)
☐	☒	For null hypothesis testing, the test statistic (e.g. *F*, *t*, *r*) with confidence intervals, effect sizes, degrees of freedom and *P* value noted<br>*Give P values as exact values whenever suitable.*
☒	☐	For Bayesian analysis, information on the choice of priors and Markov chain Monte Carlo settings
☒	☐	For hierarchical and complex designs, identification of the appropriate level for tests and full reporting of outcomes
☒	☐	Estimates of effect sizes (e.g. Cohen's *d*, Pearson's *r*), indicating how they were calculated

Our web collection on statistics for biologists contains articles on many of the points above.

Software and code

Policy information about availability of computer code

Data collection	No specific software was used for data collection.
Data analysis	Structural variants were detected in long-read sequencing data sets using the following algorithms: Sniffles2 v2.2, cuteSV v2.1.0, SVIM v1.4.2, Severus v1.0, and SVision-pro v2.1. All ONT data were aligned to GRCh38 and T2T-CHM13v2.0 reference genomes with minimap2 (v2.24) with parameters “-ax map-ont-MD”. ONT reads were phased using WhatsHap v2.3 and germline heterozygous SNPs detected using strelka-2.9.10.centos6_x86_64.<br><br>Illumina WGS data sets were aligned to the reference genome using BWA-MEM v0.7.17-r1188, and processed with the Genome Analysis Toolkit (GATK, v4.1.8.0) to remove duplicates and recalibrate base quality scores. We used NGSCheckMate v1.0.1 to verify that sequencing data from tumour-normal pairs were properly matched. SVs were identified in Illumina data using GRIDSS (v2.12.0). B-allele frequency (BAF) values were calculated using AMBER (v3.5), read depth ratios for heterozygous SNPs were computed using COBALT (v1.11). The outputs of AMBER and COBALT were used as input to PURPLE (v2.54) to estimate the purity, ploidy, and copy number aberrations in tumour samples. Microsatellite instability was assessed using PURPLE. QC statistics and plots were generated using cramino v0.14.5.<br><br>All statistical analyses were performed using R version 4.2.2. The level of significance for all statistical analyses was set at 0.05. No statistical method was used to predetermine sample size. Rearrangement and copy number profiles were visualised using the R package ReConPlot68 v0.1. Structural variants were read into R data frames for visualisation purposes using the R package StructuralVariantAnnotation n v1.14.

For manuscripts utilizing custom algorithms or software that are central to the research but not yet described in published literature, software must be made available to editors and reviewers. We strongly encourage code deposition in a community repository (e.g. GitHub). See the Nature Portfolio guidelines for submitting code & software for further information.

Data

Policy information about availability of data

All manuscripts must include a data availability statement. This statement should provide the following information, where applicable:

- Accession codes, unique identifiers, or web links for publicly available datasets
- A description of any restrictions on data availability
- For clinical datasets or third party data, please ensure that the statement adheres to our policy

WGS data from the participants enrolled in the 100,000 Genomes Project can be accessed via Genomics England Limited following the procedure described at: https://www.genomicsengland.co.uk/about-gecip/joining-research-community/. In brief, applicants from registered institutions can apply to join one of the Genomics England Research Networks, and then register a project. Access to the Genomics England Research Environment is then granted after completing online training. The short and long-read sequencing data from the glioblastoma samples are available under controlled access at EGA under the accession number EGAS50000000651. Data access can be granted via the EGA for a defined time period after successful completion of a data access agreement provided by the WTSI CGP Data access committee (datasharing@sanger.ac.uk). ONT sequencing data for the COLO829 and COLO829BL cell lines35 using R9.4 MinION/GridION flow cells were downloaded from the European Nucleotide Archive (ENA; project ID PRJEB27698). The nanopore WGS data generated by Oxford Nanopore Technologies (Oxford, UK) for the cell lines COLO829 and COLO829BL using the Ligation Sequencing Kit v14 were downloaded from the Amazon Web Services S3 bucket s3://ont-open-data/colo_2023.04/. Finally, PacBio HiFi sequencing data for COLO829 and COLO829BL generated using the Revio system were downloaded from https://downloads.pacbcloud.com/public/revio/2023Q2/COLO829/. The reference genome build GRCh38 was downloaded from https://hgdownload.soe.ucsc.edu/downloads.html. The genome assembly T2T-CHM13v2.0 was downloaded from https://www.ncbi.nlm.nih.gov/datasets/genome/GCF_009914755.1/.

Human research participants

Policy information about studies involving human research participants and Sex and Gender in Research.

Reporting on sex and gender	We did not collect gender data in this study. We provide biological sex information for all samples analysed in Supplementary Table 1. Biological sex information was not used to define experimental groups or stratify samples for any of the analyses reported.
Population characteristics	The selection of tumour samples and data sets analysed was not guided by population information. Patient demographic information, including biological sex, age and tumour diagnosis, is provided in Supplementary Table 1.
Recruitment	No specific recruitment criteria were applied for the selection of tumour samples to be sequenced except for tumour quality. Specifically, fresh-frozen tissue sections [haematoxylin and eosin (H&E); 5 µm] were used to guide the selection of the most viable areas for each tumour specimen in terms of lack of necrosis and tumour cellularity. DNA was extracted from matched tumour and blood samples using established protocols and in accordance with the 100,000 Genomes Project guidelines.
Ethics oversight	Fresh-frozen bone and soft-tissue sarcoma samples were obtained from patients consented and enrolled in both the Genomics England 100,000 Genomes Project (G100k) as well as the Royal National Orthopaedic Hospital (RNOH) Biobank, satellite of the UCL/UCLH Biobank for Health and Disease (REC reference 20/YH/0088). Patients did not receive financial compensation for donating samples.<br><br>Glioblastoma and matched blood samples were collected in the Neurosurgery Department at Centro Hospitalar Universitário Lisboa Norte (CHULN) and stored less than 1h after surgery at Biobanco-iMM CAML (Lisbon Academic Medical Center, Lisbon, Portugal). Ethical approval was obtained from the Ethics Committee of CHULN (Ref. Nº 367/18). Written informed consent was obtained from all patients prior to study participation in accordance with the European and National Ethical Regulation (law 12/2005). Patients did not receive financial compensation for donating samples.

Note that full information on the approval of the study protocol must also be provided in the manuscript.

Field-specific reporting

Please select the one below that is the best fit for your research. If you are not sure, read the appropriate sections before making your selection.

☒ Life sciences ☐ Behavioural & social sciences ☐ Ecological, evolutionary & environmental sciences

For a reference copy of the document with all sections, see nature.com/documents/nr-reporting-summary-flat.pdf

Life sciences study design

All studies must disclose on these points even when the disclosure is negative.

Sample size	No sample size calculations were performed.
Data exclusions	QC statistics and plots were generated using cramino version 0.14.5.<br>We did not exclude any data points from the published data sets analysed.

Replication	To assess the performance of SV detected algorithms we used replication experiments. Specifically, we used simulated sequencing replicates of human tumour nanopore whole-genome sequencing data sets, and sequencing replicates of the melanoma cell line COLO829BL. All replication analyses were successful.
Randomization	No specific allocation of samples was performed. Randomization was applied when we generated simulated seqeuncing replicates. Sepecifically, sequencing reads were assigned to each simulated sequencing replicate file randomly.
Blinding	The researchers involved in this study were not blinded to sample allocation across groups during data analysis as the selection of samples for sequencing was largely driven by sample availability and quality based on histophatological examination of tumour samples.

Reporting for specific materials, systems and methods

We require information from authors about some types of materials, experimental systems and methods used in many studies. Here, indicate whether each material, system or method listed is relevant to your study. If you are not sure if a list item applies to your research, read the appropriate section before selecting a response.

Materials & experimental systems

n/a	Involved in the study
☒	☐ Antibodies
☒	☐ Eukaryotic cell lines
☒	☐ Palaeontology and archaeology
☒	☐ Animals and other organisms
☒	☐ Clinical data
☒	☐ Dual use research of concern

Methods

n/a	Involved in the study
☒	☐ ChIP-seq
☒	☐ Flow cytometry
☒	☐ MRI-based neuroimaging
